# Genomic analyses suggest parallel ecological divergence in *Heliosperma pusillum* (Caryophyllaceae)

**DOI:** 10.1111/nph.14722

**Published:** 2017-08-07

**Authors:** Emiliano Trucchi, Božo Frajman, Thomas H. A. Haverkamp, Peter Schönswetter, Ovidiu Paun

**Affiliations:** ^1^ Department of Botany and Biodiversity Research University of Vienna Rennweg 14 Vienna 1030 Austria; ^2^ Department of Life Sciences and Biotechnology University of Ferrara Via L. Borsari 46 Ferrara 44121 Italy; ^3^ Institute of Botany University of Innsbruck Sternwartestraße 15 Innsbruck 6020 Austria; ^4^ Department of Biosciences Centre for Ecological and Evolutionary Synthesis University of Oslo PO Box, 1066 Blindern Oslo 0316 Norway

**Keywords:** coalescent‐based simulations, demography, phyllosphere, RAD sequencing, repeated evolution

## Abstract

- The mosaic distribution of interbreeding taxa with contrasting ecology and morphology offers an opportunity to study microevolutionary dynamics during ecological divergence. We investigate here the evolutionary history of an alpine and a montane ecotype of *Heliosperma pusillum* (Caryophyllaceae) in the south‐eastern Alps.
- From six pairs of geographically close populations of the two ecotypes (120 individuals) we obtained a high‐coverage restriction site associated DNA sequencing (RADseq) dataset that was used for demographic inference to test the hypothesis of parallel evolution of the two ecotypes.
- The data are consistent with repeated ecological divergence in *H*. *pusillum*, uncovering up to five polytopic origins of one ecotype from the other. A complex evolutionary history is evidenced, with local isolation‐with‐migration in two population pairs and intra‐ecotype migration in two others. In all cases, the time of divergence or secondary contact was inferred as postglacial. A metagenomic analysis on exogenous contaminant RAD sequences suggests divergent microbial communities between the ecotypes.
- The lack of shared genomic regions of high divergence across population pairs illustrates the action of drift and/or local selection in shaping genetic divergence across repeated cases of ecological divergence.

## Introduction

Ecologically divergent populations, eventually evolving into stable ecotypes, constitute an ideal opportunity to study the pace and mode of neutral and adaptive processes along the speciation continuum (Rundle & Nosil, 2005; Butlin *et al*., 2008; Schluter & Conte, 2009). The evolution of ecotypes is driven by two fundamentally different processes, that is selection on locally adaptive alleles and random drift, which is especially strong in small populations across fragmented landscapes. Given this complexity, repeated evolution of ecotypes provides natural replicates that add significant power to tackle fundamental evolutionary questions about the trajectory of genomic divergence from locally adapted populations to isolated species, and to infer the adaptive role of different alleles (Seehausen *et al*., 2014; Lowry, 2012).

Even if recurrent origins of morphological and/or ecological adaptation is a more common phenomenon than originally predicted (Levin, 2001; Wood *et al*., 2005), only a few cases have been documented up to now. Besides the paradigmatic case of parallel freshwater adaptation in threespine stickleback (Rundle *et al*., 2000; Colosimo *et al*., 2005), multiple origins of the same ecotype have been discovered among seaweeds (Pereyra *et al*., 2013), angiosperms (Brochmann *et al*., 2000; Berglund *et al*., 2004; Foster *et al*., 2007; Roda *et al*., 2013), invertebrates (Butlin *et al*., 2014; Soria‐Carrasco *et al*., 2014) and vertebrates (Østbye *et al*., 2006). Recurrent *de novo* evolution can result (1) from independent origins of the underlying molecular modification leading to similar phenotypes via recurrent mutations at the same genomic location, (2) through different alterations in the same gene producing a similar product or (3) through convergent changes in different components of the same molecular pathway (for a review see Stern, 2013). Alternatively, selection can act on already existing variation. Different populations of a species could be exposed to a similar selective pressure in a marginal or patchy habitat in the species range. As a consequence, any trait that is advantageous in that specific habitat and is present in the gene pool of the different populations as shared polymorphism (e.g. standing or flowing variation) will probably increase in frequency (Loh *et al*., 2013; Stankowski, 2013; Pearse *et al*., 2014; Lamichhaney *et al*., 2015; O'Brown *et al*., 2015).

A mosaic (i.e. interspersed) distribution of ecotypes, with genetic diversity rather structured by geographical proximity (isolation‐by‐distance; IBD) than by ecology (isolation‐by‐ecology; IBE), is often proposed as indirect evidence of repeated ecological divergence (e.g. Nosil *et al*., 2008; Johannesson *et al*., 2010). In such cases, a contrasting pattern of higher relatedness by ecology is expected only around ecologically relevant adaptive loci. The lack of such pattern at some genetic traits is not, however, conclusive evidence against parallel ecological divergence. In fact, different mutations at the same locus or at different loci participating towards the same adaptive trait can arise and/or be fixed in different populations independently (Pritchard *et al*., 2010; Wellenreuther & Hansson, 2016). Signal of local different adaptation could also be diluted over time by gene flow, leaving a signature (i.e. partial or soft sweeps) that is very difficult to detect (Ralph & Coop, 2010; Hermisson & Pennings, 2017). Genome‐wide signature of IBD across interspersed populations of different ecotypes could also be produced by a single origin of the ecological divergence followed by independent range expansion of the two ecotypes and subsequent intensive local introgression at different localities (Bierne *et al*., 2011, 2013; Welch & Jiggins, 2014). According to this hypothesis, gene flow is expected to homogenize the differentiation accumulated in allopatry across the whole genome but divergence will remain preserved at adaptive loci by selection. Even if secondary contacts followed by admixture and hybridization, driven for example by recurrent climatic shifts, are fundamental processes in evolution (Hewitt, 2001, 2011), the independent colonization (i.e. without any gene flow) of mosaically distributed habitats with contrasting ecology followed by a sudden re‐opening of gene flow between the two habitats at many different localities (‘instantaneous colonization’ model in Bierne *et al*., 2013) can be considered as arguably less parsimonious.

**Figure 1:**
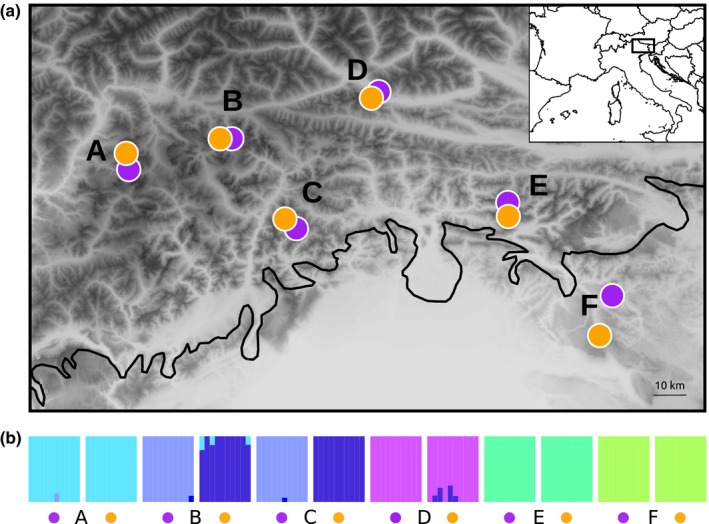
(a) Map of sampled ecotype pairs of *Heliosperma pusillum* (alpine ecotype, orange; montane ecotype, purple) in south‐eastern Alps. The extension of the ice sheet during the Last Glacial Maximum is shown with a solid black line. The inset shows the position of the sampling area in Europe. (b) Barplots showing proportion of ancestry relative to *k *=* *6 clusters as inferred in faststructure. Colors identifying each cluster are randomly assigned and used only in this figure.

An excellent model to test the hypothesis of repeated ecological divergence is provided by the perennial caespitose herb *Heliosperma pusillum* (Caryophyllaceae) in the eastern Alps where two ecotypes, alpine and montane, present a mosaic distribution (Fig. 1). These two ecotypes inhabiting rocky calcareous habitats have also been described as distinct species, namely *H. pusillum* Vis. and *H. veselskyi* Janka. The former is widely distributed from the Sierra Cantabrica in the west to the Carpathians in the east (Frajman & Oxelman, 2007), whereas the latter comprises a few disjunct populations restricted to the south‐eastern Alps, usually occurring near to alpine ecotype even if separated by forest‐covered slopes extending over several hundred meters of altitude. The alpine ecotype is characterized by glabrous or sparsely hairy leaves and occasional presence of unicellular glands (Neumayer, 1923; Frajman & Oxelman, 2007; Frajman *et al*., 2009) and occurs in damp, rocky habitats above the timberline. The montane ecotype has instead a dense indumentum with long multicellular sticky glandular trichomes and is found in canyons and below cliff overhangs with dry soils and poor light conditions far below the timberline. Phenotypic differences between the two ecotypes remain stable after cultivation under uniform conditions for at least three generations but inter‐ecotype crossings produce viable and fertile offspring (Bertel *et al*., 2016a). Available phylogenies (Frajman & Oxelman, 2007; Frajman *et al*., 2009) suggest that the relationships among populations of both ecotypes may be governed by geographic proximity rather than by ecological preference, implying that the disjunct populations of the montane ecotype could have originated more than once from the widespread alpine populations.

Using genome‐wide single nucleotide polymorphisms (SNPs) produced by restriction site‐associated DNA sequencing (RADseq) of six pairs of geographically close populations of the two ecotypes, our main aim here was to test whether the montane–alpine ecotypic differentiation is the result of a single divergence event or if it originated independently multiple times. We first characterized the observed patterns of genetic divergence, by evaluating the strength of IBD vs IBE among localities, by estimating the amount of admixture between populations, and by assessing the level of parallel genetic divergence between the two ecotypes across localities. We finally assessed the relative probability of three demographic models representing different historical processes behind the observed pattern of divergence: strict isolation, isolation‐with‐migration and secondary contact following a period of allopatric separation.

## Materials and Methods

### Sampling and RADseq library preparation

Leaf material from 10 individuals each was collected from six pairs of geographically close populations (120 individuals in total) of the alpine and montane ecotypes of *H. pusillum* (in the following termed ‘ecotype pairs’, comprising one alpine and one montane population each, and identified with uppercase letters A–F) across the distribution range of the montane ecotype (Fig. 1; Supporting Information Notes S1, Table S1). In all localities, the two ecotypes were separated by several hundred meters of altitude (between 500 and 1200 m) and linear distance (between 1.3 and 15 km), mostly occupied by forest. Sampled material from each individual was collected in the field and immediately dried in silica gel in separate bags to avoid cross‐contamination. As the plants do not propagate vegetatively, the separation of individuals was straightforward.

Single‐digest RAD libraries were prepared using the *Sbf*I restriction enzyme (New England Biolabs, Ipswich, MA, USA) and a protocol adapted from Paun *et al*. (2016) with modifications. In short, we started with 125 ng DNA per individual and ligated 125 mM P5 barcoded adapters to the restricted samples overnight at 16°C. Shearing by sonication was performed with a Bioruptor Pico (Diagenode, Seraing, belgium) with two cycles of 45 s ‘on’ and 60 s ‘off’ at 6°C, targeting a size range of 200–800 bp. Four libraries of individually barcoded samples were finally sequenced on an Illumina HiSeq at VBCF (http://www.vbcf.ac.at/facilities/next-generation-sequencing/) as 100 bp paired‐end reads.

### Identification of RAD loci and SNP calling

The raw reads were quality filtered and demultiplexed using *process_radtags.pl* included in the Stacks1.19 package (Catchen *et al*., 2013). As medium to large plant genomes largely consist of transposable elements (TEs), which may interfere with the locus‐by‐locus assembly in the Stacks analysis, we checked for the presence of TEs in the raw reads. For this, we Blasted all paired‐end reads to the RepBase database of plant TEs from the Giri repository (http://www.girinst.org/). Each individual sample was analyzed independently and the abundance of TEs was compared among populations and between species (Notes S2; Figs S1, S2). However, as *Sbf*I has a recognition site with 75% GC content, we expected a generally low representation of TEs, which are rather AT‐rich.

The 95‐bp‐long RAD loci were further assembled and SNPs were called using *denovo_map.pl* in Stacks. A preliminary dataset was built using a minimum coverage to identify a stack (−*m*) of 20×, a maximum number of differences between two stacks in a locus in each sample (−*M*) of seven, and a maximum number of differences among loci to be considered as orthologous across multiple samples (−*n*) of nine. In this dataset, a bimodal distribution of the GC content across loci was discovered, with one peak below 45% GC and a second one above 60% (Notes S3; Fig. S3a). We suspected the latter to be due to exogenous bacterial DNA contamination, expected to result in a relatively high number of loci present in only a few individuals and having much lower coverage than the loci of the target organism. We further used a metagenomic approach on this dataset to identify microbiome contamination (see Notes S4; Figs S4–S6; Table S2). We then increased our coverage threshold for a locus to be assembled in each individual sample by setting the −*m* parameter in *denovo_map.pl* to 100. Results then showed a unimodal distribution of GC content peaking at 40–45% (Fig. S3a).

The function *export_sql.pl* in Stacks was used to extract loci information from the catalog filtering for a maximum number of missing samples per locus of 67% and a maximum number of SNPs per locus of 10. Custom Python scripts were used to further filter the loci for downstream analyses. A locus was discarded if (1) the proportion of heterozygous individuals was > 0.65, an arbitrary cut‐off used to reduce the risk of including paralogs; (2) the GC content was > 65%, to further minimize the risk of including bacterial sequences; (3) any of the samples was scored as triallelic, again reducing the risk of including paralogs in the dataset; or (4) it was invariant. We observed an increase in the occurrence of SNPs in the last 10 positions of the reads (Fig. S3b). As we could not assess their validity, we discarded all SNPs found in this portion of the reads. To check for the proportion of loci containing coding regions, filtered loci were Blasted against the NCBI *nt* database and an available *Silene vulgaris* transcriptome (http://silenegenomics.biology.virginia.edu/; Sloan *et al*., 2012).

### Structure of genetic diversity

Summary statistics of genetic diversity were estimated in each population using the final common catalog of loci (1097 variable loci, containing 3401 SNPs): expected heterozygosity (*H*_e_), individual observed heterozygosity normalized by coverage (*H*_o ind_; Trucchi *et al*., 2016), Watterson's θ and π.

To assess the effect of distance among localities on genetic divergence, we tested for IBD across all populations by comparing matrices of genetic and geographic distances. Nei's genetic distances among localities, including a sample size correction factor (Nei, 1978), were estimated using the gstudio package in R (Dyer & Nason, 2004). In addition, we tested the effect of IBE (alpine vs montane ecology) on the genetic structure by comparing matrices of ecological (converted to a binary factor) and genetic distance. To assess statistical correlation among matrices we first applied Mantel tests with 9999 randomizations between geographic and genetic distance and between ecological and genetic distance. We then applied a partial Mantel test (*mantel.partial* function in vegan R package; Oksanen *et al*. 2013) with 9999 randomizations between ecological and genetic distance but controlling for geographic distance. In addition, using the *dist_amova* function in the gstudio R package and the *adonis* function in the vegan R package, a multilocus MANOVA was used to test for statistical differentiation among populations (i.e. 12 groups), ecotype pairs (i.e. six groups) and ecotypes (i.e. two groups).

To assess the level of parallel genetic divergence between the two ecotypes across localities we applied a hierarchical approach as presented by Ravinet *et al*. (2016) identifying highly divergent loci in each ecotype pair and then comparing the co‐occurrence of such highly divergent loci in multiple ecotype pairs with a null‐expectation based on random overlap. Using the information provided by all linked SNPs at each 95‐bp locus, we first searched for highly divergent loci in each ecotype pair applying a coalescent‐based method (Beaumont & Nichols, 1996). lositan (Antao *et al*., 2008) was used to perform 500 000 coalescent simulations and produce joint distributions of *F*_ST_ and expected heterozygosity in a simple island model without selection for each ecotype pair. The simulations aimed at the observed mean *F*_ST_ value estimated for each ecotype pair. The observed loci with joint distribution of *F*_ST_ and expected heterozygosity above the 99^th^ quantile (false discovery rate (FDR) = 0.01) were considered as local highly divergent loci. The six lists of such loci (one for each ecotype pair) were compared to each other looking for shared highly divergent loci among two or more ecotype pairs. None of these highly divergent loci was shared by five or more ecotype pairs. Using the exact number of screened loci and the number of highly divergent loci scored in each ecotype pair, we simulated (by 1000 randomizations) the expected random distribution of shared loci for each comparison. Observed values were then compared with their expected random distribution. In the case of more overlap between ecotype pairs than expected by chance, we investigated the contribution of each ecotype to this signal by comparing the differentiation within one ecotype to the differentiation within the other ecotype. For this analysis we plotted the joint distribution of between‐localities intra‐ecotype *F*_ST_ (see Fraïsse *et al*., 2016).

To investigate the level of admixture among populations we first used the *find.clusters* function in the R package adegenet (Jombart & Ahmed, 2011), which estimates the likelihood of structure in the sample according to a range of clusters (here k‐means analysis ranged from 1 to 20). A Bayesian information criterion (BIC) is applied to choose the best *k*. The Bayesian algorithm implemented in faststructure (Raj *et al*., 2014) was then used to estimate the cluster's contribution in each individual. As only independent biallelic loci are allowed, we selected one SNP at random from each RAD locus. The results of population structure analysis were plotted using python. The structure of genetic diversity across all individuals was further investigated by principal component analysis (PCA) using the *glPca* function in the R package adegenet (Jombart & Ahmed, 2011). Individuals from A, B, C and D were also analyzed separately. To reconstruct the relationships among populations of the two ecotypes taking into account admixture, we used treemix (Pickrell & Pritchard, 2012). The result of this analysis includes a maximum‐likelihood (ML) tree of population ancestry, showing also estimated migration events. For the input file, SNP data were converted from diploid genotype calls for each individual into population‐level allele counts using a custom python script (Notes S5). Migration edges were progressively added to the tree and exit log‐likelihood and Akaike's Information Criterion (AIC) were recorded.

### Testing alternative demographic scenarios of local divergence

The demographic scenario underlying the observed pattern of local divergence was investigated in the four population pairs for which plain local divergence of the two ecotypes has been inferred in our previous analyses (i.e. A, D–F). Pairs B and C were excluded from this analysis as it became clear from treemix and faststructure results that they have not simply diverged locally from one another. We compared three different demographic models: (1) strict isolation with no gene flow (SI), (2) isolation‐with‐migration (IM) and (3) secondary contact after allopatry (SC). We used a composite‐likelihood maximization method to estimate the best likelihood by simulating joint site frequency spectra under a continuous‐time Markovian coalescent model in fastsimcoal2 (Excoffier *et al*., 2013). For all models, we let the algorithm estimate the effective size of each population (*N*_a_ and *N*_m_ for the alpine and montane ecotype, respectively; ancestral *N* was rescaled on *N*_a_) and the mutation rate (μ). Substitution rate is estimated by using an observed site frequency spectrum based on one selected SNP per 95‐bp locus and the resulting value needs to be scaled by two orders of magnitude to represent an average genome‐wide substitution rate. Migration rates in both directions (*m*_am_ and *m*_ma_) were estimated in the IM and SC models, time since divergence (*T*_d_) of the two ecotypes in SI and IM, and time since secondary contact (*T*_c_) between the two ecotypes in SC. In the last, the time of the initial divergence was fixed to 50 000 generations ago, before the last Würm glaciation peak (Ivy‐Ochs *et al*., 2008). Generation time in this species can be considered 1 yr. After excluding the SNP potentially under selection, the folded two‐dimensional site frequency spectrum (2D‐SFS) for each of the four ecotype pairs was used as summary statistic selecting one random SNP per locus (Notes S6; Fig. S7). For each locus, the global frequency of the two alternative alleles across all investigated populations was used to determine the minor allele (i.e. probably the derived one) before estimating its frequency (normalized for missing data) at a local scale. Hence, a minor allele can have a frequency > 0.5 in a specific population (Fig. S7). As variable loci were selected to be present across all populations, invariant loci in both populations of any ecotype pair were the most represented category in the 2D‐SFS. To overcome this issue and further validate our results, analyses were overrepresented in the 2D‐SFS of each pair for each ecotype pair separately. fastsimcoal2 was run without using fixed sites in the spectrum (−0 option). We performed a maximum of 60 expectation/conditional maximization (ECM) cycles with each step requiring the generation of 100 000 simulated joint‐spectra. We ran 10 replicates of each model for each ecotype pair. Convergence of ML and parameter estimates was checked and different models were then compared using AIC.

We further tested the goodness‐of‐fit of the combination ‘scenario + posterior estimates of the parameters’ to the data generating pseudo‐observed datasets and comparing them with the observed datasets through summary statistics (see Bertorelle *et al*., 2010 for applications of this test in Approximate Bayesian Computation). Average values of *N*_a_, *N*_m_, *m*_am_, *m*_ma_, μ, and *T*_d_ or *T*_c_ estimated above were used in pseudo‐observed dataset simulations for each ecotype pair. We used π as a summary statistic. For each ecotype pair, we simulated 1000 100‐bp‐long loci on which we estimated the distribution of π calculated on all individuals of both ecotypes (combined π) under the IM and the SC models. The statistical difference between the simulated distribution of π for each model and the observed distribution was assessed by a Kolmogorov–Smirnov two‐samples test.

We then estimated the 95% confidence intervals of the estimates of the demographic parameters in those models passing both the AIC and the goodness‐of‐fit criteria. For each of the successful models, we used the point estimates of the best‐likelihood run to simulate 100 2D‐SFS, which were then used to re‐estimate the demographic parameters through 30 likelihood‐maximization runs each. These 100 estimates were then used to build the 95% confidence intervals for each parameter in each of the selected models (see also https://groups.google.com/forum/#!topic/fastsimcoal/N956Af31iA4).

### Data accessibility

Data and additional results are available from the Dryad Digital Repository (doi: 10.5061/dryad.391q5). Raw genomic data are available from the NCBI Short Reads Archive (accession nos. SRP065672, SRP068291).

## Results

### Genomic datasets

The average number of raw pairs of reads per sample retained after quality filtering was 2.1 (SD = 0.8) million. The proportion of paired‐end reads Blasting to the Viridiplantae Transposable Elements database was very low for all individuals, 0.5–4.1%, with a higher proportion (two‐samples Kolmogorov–Smirnov *P* = 0.047) in the montane ecotype (Notes S2). The bacterial and fungal contamination in our dataset was significantly different between the two ecotypes (PERMANOVA *P* = 0.002; Notes S4) with most of the fungal taxa and the bacteria Xanthomonadales occurring across most of, but only in, the montane populations (Table S2; Fig. S5).

After *de novo* catalog building and SNP calling, we selected 1719 high‐coverage loci present in at least 40 samples. A total of 172 invariant loci were discarded. Average coverage per allele was *c*. 300× in the alpine individuals and *c*. 200× in the montane ones (Fig. S3a). The lower coverage in the trichome‐rich montane ecotype was due to higher exogenous DNA contamination. It was possible to map 36% of the retained loci to the *S. vulgaris* transcriptome and an additional 5% to coding sequences of other plant species in the NCBI database. Four of the loci matched two fungal sequences (poplar leaf fungus), one bacterial sequence (*Borrelia*) and one nematode sequence (Ascaridae). These loci were removed from the dataset. We further filtered the final dataset according to the criteria detailed in the Methods section to a total of 1097 variable loci, containing 3401 SNPs and occurring on average in 102 individuals (Fig. S3b,c).

### Structure of genetic diversity

Expected heterozygosity (*H*_e_), Watterson's θ and π for each population of the two ecotypes are given in Table S3 (Notes S7). Boxplots of observed heterozygosity (*H*_o ind_) comparing the two ecotypes at each locality are shown in Fig. S8.

The IBD model (Fig. S9) was confirmed (*r *=* *0.69, *P *=* *0.0002) whereas IBE had no effect on the genetic structure (Mantel test ecological vs genetic distance: *r *=* *−0.08, *P *=* *0.89; partial Mantel test ecological vs genetic distance controlling for geographical distance: *r *=* *0.01, *P *=* *0.34). The three alternative MANOVAs indicated that the most variance is explained by differences between populations, or between ecotype pairs, but not between the two ecotypes (differentiation among 12 populations: *R* ^2^ = 0.91, *P *=* *0.001; six ecotype pairs: *R* ^2^ = 0.83, *P *=* *0.001; two ecotypes: *R* ^2^ = 0.02, *P *=* *0.14).

**Figure 2:**
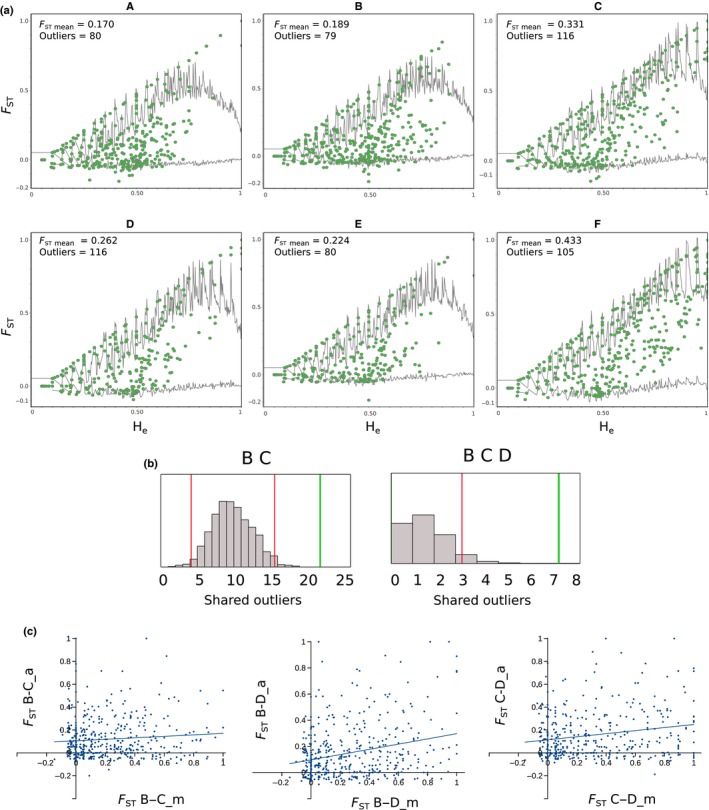
Shared highly divergent loci across six pairs of the alpine and the montane ecotype of *Heliosperma pusillum*. (a) Joint distribution of *F*_ST_ and expected heterozygosity (*H*_e_) across all loci (i.e. haplotypes) between the two ecotypes in each pair. Average *F*_ST_ and number of highly divergent loci in each ecotype pair are indicated. Green dots: observed loci; gray lines: upper and lower bounds of the joint distribution of *F*_ST_ and *H*_e_ estimated by 500 000 coalescence‐based simulations. (b) Significant overlap of highly divergent loci is found between B and C and among B, C and D. Gray bars, null distribution of shared highly divergent loci (1000 randomizations) under neutrality; red vertical lines, 95% quantiles of the null distribution; green vertical lines, observed number of shared highly divergent loci. See Figs S9–S11 for all possible comparisons with two, three and four ecotype pairs. (c) Joint distribution of *F*_ST_ between the alpine and the montane ecotypes between ecotype pairs with significantly over‐shared highly divergent loci.

Analyzing the six ecotype pairs separately with lositan, we found between 79 and 116 loci with an extreme *F*_ST_ (FDR = 0.01) taking into account 500 000 simulations of the joint distribution of *F*_ST_ and *H*_e_ (Fig. 2a). Ecotype pair mean *F*_ST_ ranged from 0.17 in A to 0.43 in F. The number of highly divergent loci shared among different ecotype pairs was not different from random expectations (Notes S8; Figs S10–S12), except for the overlap between B and C, and among B, C and D (Fig. 2b). The joint *F*_ST_ distribution between montane B vs C populations and alpine B vs C populations (Fig. 2c) revealed a much higher differentiation between the montane populations than between the alpine ones (slope of linear regression = 0.06, *R* ^2^ = 0.009). The same was found in the comparison B vs D and C vs D (slope of linear regression = 0.13 and 0.2, *R* ^2^ = 0.04 and 0.07, respectively). At the global level (Fig. S13), the differentiation among montane populations was slightly stronger than among the alpine ones (slope of linear regression = 0.44, *R* ^2^ = 0.2).

**Figure 3:**
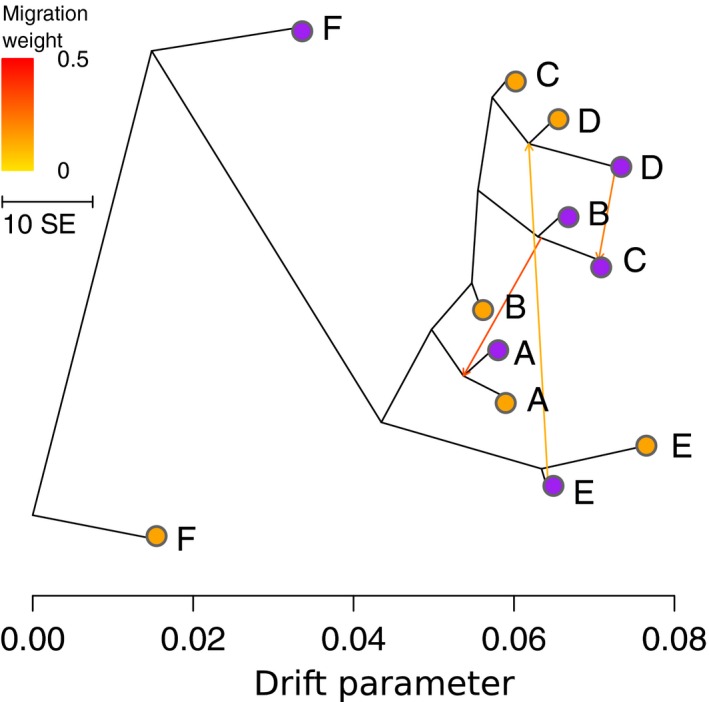
Maximum‐likelihood tree and migration events among six pairs of the alpine and the montane ecotype of *Heliosperma pusillum*. Population tree and migration edges have been inferred by treemix. Orange, alpine ecotype; purple, montane ecotype; population labels as in Fig. 1.

BIC in *find.clusters* analysis started plateauing after *k *=* *6 (Fig. S14). Few individuals with putatively admixed ancestry were found by faststructure in populations A, B, C and D (Fig. 1). PCAs clearly clustered individuals by locality and not by ecology with the exception of populations B and C (Fig. S15). The topology of the ML population tree reconstructed with treemix (Fig. 3) corresponded to the structure inferred by faststructure and PCA. After adding more than three migration edges the likelihood reached a plateau.

### Testing alternative demographic scenarios

**Figure 4:**
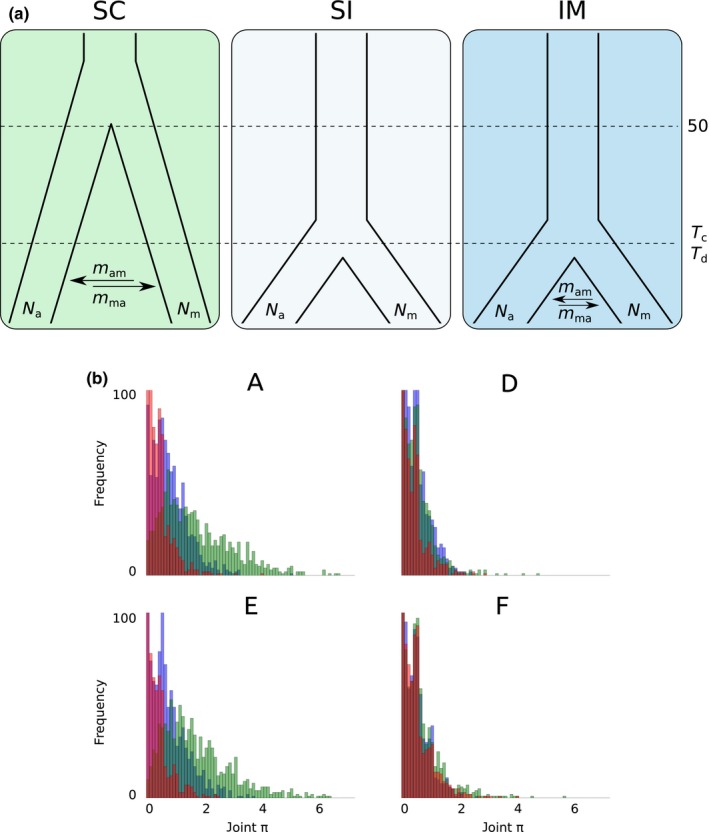
Demographic scenario potentially leading to local divergence between the alpine and the montane ecotype in *Heliosperma pusillum*. (a) Three alternative demographic models are compared: allopatric divergence followed by secondary contact (SC), strict isolation with no gene flow (SI) and isolation‐with‐migration (IM). Effective population size of the montane and the alpine ecotype (*N*_a_ and *N*_m_), migration rates in both directions (*m*_am_ and *m*_ma_) and time since onset of gene flow (*T*_c_) or since the split (*T*_d_) have been estimated in fastsimcoal2 using the two‐dimensional site frequency spectrum (2D‐SFS) in each ecotype pair. The SD model was rejected in all cases. (b) Goodness‐of‐fit of the combination ‘scenario + posterior estimate of the parameters’ for SC and IM have been further assessed by pseudo‐observed dataset simulation using the distribution of combined π as the summary statistic. Maximum frequency has been cut to 100 for graphical clarity. In ecotype pairs A and E, the SC model was rejected. See the text for Kolmogorov–Smirnov test results. Green bars, SC model; purple bars, IM model; red bars, observation.

In all tested ecotype pairs, the scenario of local divergence with no migration (SI) was rejected, whereas the isolation‐with‐migration (IM) and the secondary contact (SC) scenarios were not distinguishable due to too similar AIC scores (Table 1). In all cases, the time of the split between the two ecotypes was estimated after the Last Glacial Maximum (the oldest split is estimated in F between 4651 and 19 189 yr ago (95% confidence intervals) according to the IM model) and the mutation rate, estimated on one selected SNP per 95‐bp locus, was *c*. 5 × 10^−7^ substitutions per site per generation. Higher migration rate from the alpine to the montane ecotype was estimated in E whereas higher rates in the other direction were inferred in D and F (Table 1). Further comparing the combined distribution of π in each ecotype pair as simulated under the two best‐fit models (SC and IM) fixing the parameters to the average ML estimates (Fig. 4; pseudo‐observed dataset simulation), the IM scenario obtained a higher goodness‐of‐fit in the case of A (Kolmogorov–Smirnov, Obs − SC_pods_: *P* = 4.3 × 10^−5^, Obs − IM_pods_: *P* = 0.55) and E (Kolmogorov–Smirnov, Obs − SC_pods_: *P* = 1.6 × 10^−6^, Obs − IM_pods_: *P* = 0.33) ecotype pairs. As the SI model was within four AIC points from the best‐fit model (SC) in E, we generated a pseudo‐observed SI dataset and, after assessing its goodness‐of‐fit as above, we definitely discarded this model (Kolmogorov–Smirnov, Obs − SI_pods_: *P* = 0). In the remaining ecotype pairs (D and F), the distribution of combined π produced under the two competing models was indistinguishable from the observation (Fig. 4). Replicated analyses building the 2D‐SFS in each ecotype pair separately, thus using a higher number of variable loci and a lower proportion of missing data, fully confirmed these results.

**Table 1:** Demographic inference for each model tested with fastsimcoal2 in four population pairs of the alpine and the montane ecotypes of *Heliosperma pusillum*

	Model	*L*	AIC	Fit	*N*_a_ (ind)	*N*_m_ (ind)	Time (yr)	*m*_ma_ (% of *N*_a_)	*m*_am_ (% of *N*_m_)	μ* (substitutions per site per generation)
A	IM	−1011	2032	Yes	110–9746	47–4321	3841–14 044	2.0e‐5–4.5e‐3	2.1e‐4–1.8e‐2	1.2e‐7–8.1e‐7
	SI	−1021	2050	–						
	SC	−1010	2032	No						
D	IM	−732	1476	Yes	102–887	646–5633	3276–14 104	3.9e‐4–4.1e‐3	1.2e‐5–4.5e‐4	2.1e‐7–8.6e‐7
	SI	−747	1501	–						
	SC	−732	1476	Yes	144–743	852–4444	3467–14 263	6.6e‐4–2.8e‐3	1.5e‐5–1.7e‐4	7.0e‐8–8.7e‐7
E	IM	−1052	2116	Yes	103–22 761	13–2135	3996–15 503	1.2e‐5–1.0e‐3	2.7e‐4–5.2e‐2	1.4e‐7–9.3e‐7
	SI	−1055	2117	–						
	SC	−1050	2113	No						
F	IM	−1388	2789	Yes	102–1718	223–3990	4651–19 189	8.3e‐5–1.7e‐3	3.4e‐5–7.4e‐4	1.3e‐8–8.9e‐7
	SI	−1416	2840	–						
	SC	−1388	2787	Yes	105–610	374–2182	3378–12 560	3.2e‐4–1.6e‐3	2.4e‐5–4.6e‐4	1.8e‐7–8.6e‐7

Mean values of likelihood (*L*) and Akaike Information Criterion (AIC) across 10 maximum‐likelihood runs for each model are given in the respective columns. Summary results (i.e. yes/no passing the criterion) of goodness‐of‐fit of the combination ‘scenario + posterior estimates of the parameters’ is reported in the column ‘fit’ (see Fig. 4 and the Results section). The 95% confidence intervals for the demographic parameters are reported for the models passing both the AIC and the goodness‐of‐fit criteria. *N*_a_, effective population size of the alpine ecotype; *N*_m_, effective population size of the montane ecotype; *m*_am_, migration rate from the alpine to the montane ecotype within ecotype pair; *m*_ma_, migration rate from the montane to the alpine ecotype within ecotype pair; Time, time since split or secondary contact; μ*, mutation rate (estimated selecting one variable SNP in each 95‐bp‐long locus); IM, isolation‐with‐migration; SI, strict divergence; SC, secondary contact.

## Discussion

### Demographic scenarios underlying the mosaic distribution of *H. pusillum* ecotypes

Using genome‐wide molecular data of 120 individuals from 12 populations we show here that the alpine and montane ecotypes of *H*. *pusillum*, usually classified as distinct species (*H. pusillum* Vis. and *H. veselskyi* Janka; Frajman & Oxelman, 2007; Fischer *et al*., 2008), are not independent evolutionary units and their populations cluster more often by locality than by ecology (Figs 1, 3, S15). Despite clear parallel differences in anatomical, morphological and ecological traits (Bertel *et al*., 2016b,c) and in the phyllosphere biotic community (Table S2; Fig. S5), genome‐wide RADseq loci presented a divergence pattern rather shaped by neutral processes (i.e. IBD, hierarchical population structure and local drift) than by similar selective environments (i.e. IBE). The retrieved mosaic distribution suggests up to five repeated instances of ecological divergence between the six alpine and montane population pairs analyzed here. Recent genomic investigations suggested parallel ecological divergence as common to several organisms (e.g. Hohenlohe *et al*., 2010; Roda *et al*., 2013; Butlin *et al*., 2014; Soria‐Carrasco *et al*., 2014; Feulner *et al*., 2015; Rougemont *et al*., 2017). In the case of *H. pusillum*, coalescent simulations suggested a scenario of isolation‐with‐migration (IM) in two out of four instances of local divergence of ecotype pairs (Fig. 4). In the other two locally diverging pairs, the two best‐fit scenarios (IM and secondary contact after allopatry, SC) had very similar likelihoods (Table 1). In these two cases, observed distributions of combined π (Fig. 4) are compatible with either a recent IM scenario or late SC even in the case of the putatively oldest split in F (the only pair currently found outside the maximum ice sheet extension at the LGM; Ivy‐Ochs *et al*., 2008). A scenario of strict isolation without gene flow has been discarded in the four ecotype pairs tested here. Migration, even if at a low level (Table 1), appears as an important component to be taken into account in the evolution of the alpine and montane ecotypes of *H. pusillum*. Indeed, in all populations, π was higher than Watterson's θ (Table S3), suggesting migration between diverging populations as an important process in this biological system.

The time of divergence estimated in each ecotype pair (Table 1) consistently postdates the last glaciation (Ivy‐Ochs *et al*., 2008). In the isolation‐with‐migration scenario, ecological divergence could have been triggered by the rapid spread of forests induced by Holocene warming (e.g. Magri *et al*., 2006), separating montane stands below overhanging cliffs from alpine populations. Globally, the structure among the populations of the two ecotypes reflects a clear IBD pattern (Fig. S9). This may be related to the limited time elapsed since the colonization of recently de‐glaciated mountains or to gene flow maintaining genetic contiguity. Indeed, some traces of admixture or – alternatively, but difficult to disentangle – shared ancestry (i.e. incomplete lineage sorting) between the alpine populations are visible across the western localities, as supported by the low differentiation (expressed as drift parameter in treemix analysis) between B, C and D, in particular (Fig. 3). In addition, the higher than expected proportion of shared outliers among these localities (Fig. 2b) is probably due to a lower differentiation between the alpine populations, as also supported by the joint distribution of *F*_ST_ analysis (Fig. 2c). On average, differentiation is lower among all alpine populations when compared with the montane ones (Fig. S13). This can be a consequence of the insular, disjunct habitat preferred by the montane ecotype, whereas the alpine populations are more continuously distributed (Poldini, 2002; Wilhalm *et al*., 2014).

### Local drift and lack of shared genetic divergence

The consistent phenotypic divergence between alpine and montane ecotypes of *H. pusillum* across the six population pairs is not paralleled by shared genetic divergence within the investigated portion of the genome (i.e. no IBE). In the majority of cases, we did not identify more loci with high *F*_ST_ shared among pairs than expected by chance (Figs 2, S10–S12). Our result comparing six ecotype pairs suggests caution when interpreting patterns of divergent selection at some loci shared by one or very few contrasting ecotype pairs. In fact, the stochastic outcome of neutral processes, such as drift, could be misinterpreted as the results of a similar selective regime. An investigation of the null expectation as proposed in Fig. S10 together with the reconstruction of the general relationships among the different populations (Fig. 3) should help to assess the false discovery rate in allelic frequency covariation analyses (Figs 3b, S10). In very few cases, highly divergent loci in the *F*_ST_−*H*_e_ scans were more frequent in two to three ecotype pairs than expected by chance, as among B, C and D (Fig. 2b). However, these populations were also characterized by a clear global signature of higher relatedness within ecotype (Figs 2c, 3), suggesting demographic processes such as within‐ecotype gene flow to be a more likely explanation than convergent adaptation due to a similar selective regime. The signature of the latter is, in fact, expected to be found only at adaptive alleles and not spread across the genome. Note, however, that heterogeneity in gene flow along the genome could produce correlated signatures of divergence at certain loci across ecotype pairs. Such signatures could be misinterpreted as the result of a similar selective process independently occurring in different populations (Le Moan *et al*., 2016; Rougemont *et al*., 2017).

A lack of a signal of shared genomic divergence across ecotype pairs has also been reported in other biological systems (e.g. Perrier *et al*., 2013; Roda *et al*., 2013; Renaut *et al*., 2014). Interestingly, investigating three pairs of wave and crab ecotypes of the rough periwinkle snail on the Swedish coast, a proportion of shared high *F*_ST_ loci similar to that found in our study has been scored by a similar analytical approach (Ravinet *et al*., 2016). Independent and different mutations occurring in the same gene, or polygenic traits underlying ecological adaptations, could explain the lack of shared genomic differentiation. The short divergence time estimated in each *Heliosperma* ecotype pair renders a significant contribution of novel mutations unlikely. Hence, repetitive divergence from standing genetic variation appears more probable (i.e. collateral evolution by shared ancestry; Barrett & Schluter, 2008; Schluter, 2009; Stankowski, 2013; Stern, 2013). However, as adaptive traits are probably polygenic (Pritchard *et al*., 2010), the signature of selection may be difficult to detect unless more advanced analytical approaches, not applicable on our data type, are used (Daub *et al*., 2013).

More importantly, the reduced representation of our RADseq dataset screened only a limited portion of the genome (0.1%) and of the genes (*c*. 3.5% – assuming *c*. 20 000 genes in the 2.2 Gb *Heliosperma* genome and *c*. 700 RAD loci mapping to the *S. vulgaris* transcriptome and gene sequences in GenBank). It is thus very likely that adaptive loci, or neutral loci linked to it, were not covered in our genome scan. In addition, if selection is acting on standing genetic variation present in the ancestral population (Pritchard *et al*., 2010), the signature of divergence around loci under selection is expected to be minimal (Hermisson & Pennings, 2005). These aspects could then exacerbate the difficulties in finding adaptive loci that, in some cases, have been shown to be restricted to a single SNP (e.g. O'Brown *et al*., 2015).

Different demographic processes (i.e. multiple divergence events and/or independent secondary contacts), probably characterize the evolutionary history of contrasting ecotypes at medium to large geographic scales (e.g. Roda *et al*., 2013; Rougemont *et al*., 2017). At the local scale and if effective population size is small, drift can largely overwhelm the mark(s) left by selection. In addition, different instances of contrasting ecotypes could be affected by a diverse range of biotic (e.g. phyllosphere communities, Fig. S5; Table S2; pathogen load, Feulner *et al*., 2015) and abiotic (e.g. microclimatic conditions; Bertel *et al*., 2016b) factors of local selection. Further studies involving whole transcriptome and/or genome sequencing are needed to specifically pinpoint the molecular components underlying this iterative ecological divergence and to fully understand the evolutionary forces driving it.

## Author contributions

B.F., P.S. and O.P. planned and designed the research. B.F. and P.S. conducted fieldwork. O.P. performed experiments. E.T. analysed data. E.T. and T.H.A.H. performed metagenomic analyses. E.T., P.S. and O.P. wrote the manuscript, and all authors commented on the manuscript.

## Supporting information

Please note: Wiley Blackwell are not responsible for the content or functionality of any Supporting Information supplied by the authors. Any queries (other than missing material) should be directed to the *New Phytologist* Central Office.

**Fig. S1** Proportion of transposable elements in the RADseq dataset.
**Fig. S2** Ecotype‐specific proportion of transposable elements.
**Fig. S3** RADseq dataset quality control.
**Fig. S4** Proportion of loci assigned to putative phyllosphere and contaminant taxa.
**Fig. S5** Analysis of putative phyllosphere and contaminant taxa by ecotype.
**Fig. S6** Analysis of putative phyllosphere and contaminant taxa by locality.
**Fig. S7** Minor 2D‐SFS estimated in each ecotype pair.
**Fig. S8** Individual observed heterozygosity normalized by coverage.
**Fig. S9** Analysis of isolation‐by‐distance.
**Fig. S10** Highly divergent loci shared among two ecotype pairs.
**Fig. S11** Highly divergent loci shared among three ecotype pairs.
**Fig. S12** Highly divergent loci shared among four ecotype pairs.
**Fig. S13** Joint distribution of *F*_ST_ between alpine and montane populations.
**Fig. S14** Bayesian Information Criterion *k*‐means analysis.
**Fig. S15** Principal component analysis.
**Table S1** Sample design
**Table S2** Microbiome structure and diversity
**Table S3** Genetic diversity
**Notes S1** Sampling.
**Notes S2** Transposable elements.
**Notes S3** Dataset quality control.
**Notes S4** Analysis of contamination by leaf microbiome.
**Notes S5** Custom Python code.
**Notes S6** Testing alternative demographic scenarios.
**Notes S7** Summary statistics of genomic diversity.
**Notes S8** Structure of genetic diversity.
