# Supplementary material for: Genomic analyses suggest parallel ecological divergence in Heliosperma pusillum (Caryophyllaceae)
Source: New Phytol. 2017 Aug 7;216(1):267–78. doi: 10.1111/nph.14722 (PMC5601199; doi:10.1111/nph.14722)
Supplement: Supplementary file 1 — Fig. S1 Proportion of transposable elements in the RADseq dataset. Fig. S2 Ecotype‐specific proportion of transposable elements. Fig. S3 RADseq dataset quality control. Fig. S4 Proportion of loci assigned to putative phyllosphere and contaminant taxa. Fig. S5 Analysis of putative phyllosphere and contaminant taxa by ecotype. Fig. S6 Analysis of putative phyllosphere and contaminant taxa by locality. Fig. S7 Minor 2D‐SFS estimated in each ecotype pair. Fig. S8 Individual observed heterozygosity normalized by coverage. Fig. S9 Analysis of isolation‐by‐distance. Fig. S10 Highly divergent loci shared among two ecotype pairs. Fig. S11 Highly divergent loci shared among three ecotype pairs. Fig. S12 Highly divergent loci shared among four ecotype pairs. Fig. S13 Joint distribution of F ST between alpine and montane populations. Fig. S14 Bayesian Information Criterion k‐means analysis. Fig. S15 Principal component analysis. Table S1 Sample design Table S2 Microbiome structure and diversity Table S3 Genetic diversity Notes S1 Sampling. Notes S2 Transposable elements. Notes S3 Dataset quality control. Notes S4 Analysis of contamination by leaf microbiome. Notes S5 Custom Python code. Notes S6 Testing alternative demographic scenarios. Notes S7 Summary statistics of genomic diversity. Notes S8 Structure of genetic diversity. [file NPH-216-267-s001.pdf]

Genomic Analyses Suggest Parallel Ecological Divergence in *Heliosperma pusillum* (Caryophyllaceae)

Emiliano Trucchi, Božo Frajman, Thomas HA Haverkamp, Peter Schönswetter, Ovidiu Paun

Article acceptance date: 20 June 2017

The following Supporting Information is available for this article

Notes S1. Sampling

Table S1. Sample design (labels as in Fig. 1 in the main text).

Population, Ecotype, Bioproject Accession, Sample ID, SRA accession, Locality, Lat_Lon, Altitude, Collected by, Collection date

A, alpine, PRJNA308183-PRJNA300879, PVA3-PVA4-PVA5-PVA6-PVA7-PVA8-PVA9-PVA11-PVA14-PVA18, SRR2891253-SRR2891254-SRR2891255-SRR2891235-SRR3096515-SRR2891236-SRR2891249-SRR2891250-SRR2891251-SRR2891252, Italy: Trentino-Alto Adige: Dolomiti di Gardena/Grödner Dolomiten, 46.601 N 11.768 E, 2290, Ruth Flatscher, 24-Jul-2011

A, montane, PRJNA308183-PRJNA300879, VVA4-VVA6-VVA12-VVA15-VVA16-VVA19-VVA20-VVA23-VVA26-VVA29, SRR2891237-SRR2891238-SRR2891239-SRR2891243-SRR2891244-SRR2891245-SRR2891246-SRR2891247-SRR3096516-SRR2891256, Italy: Trentino-Alto Adige: Dolomiti di Gardena/Grödner Dolomiten, 46.564 N 11.77 E, 1690, Ruth Flatscher, 24-Jul-2011

B, alpine, PRJNA308183, PTO5-PTO6-PTO8-PTO12-PTO19-PTO2-PTO20-PTO22-PTO25-PTO27, SRR3096551-SRR3096552-SRR3096557-SRR3096553-SRR3096554-SRR3096555-SRR3096556-SRR3096558-SRR3096559-SRR3096560, Italy: Trentino-Alto Adige: Dolomiti di Braies/ Pragser Dolomiten,, 46.644 N 12.205 E, 2190, Ruth Flatscher, 8-Jul-2011

B, montane, PRJNA308183, VTO8-VTO10-VTO11-VTO14-VTO16-VTO21-VTO23-VTO27-VTO28-VTO29, SRR3096614-SRR3096615-SRR3096616-SRR3096617-SRR3096618-SRR3096619-SRR3096620-SRR3096621-SRR3096622-SRR3096623, Italy: Trentino-Alto Adige: Dolomiti di Braies/ Pragser Dolomiten,, 46.645 N 12.233 E, 1420, Ruth Flatscher, 8-Jul-2011

C, alpine, PRJNA308183, PCI15-PCI16-PCI17-PCI18-PCI19-PCI20-PCI22-PCI23-PCI24-PCI30, SRR3096512-SRR3096513-SRR3096527-SRR3096538-SRR3096550-SRR3096561-SRR3096572-SRR3096583-SRR3096601-SRR3096613, Italy: Friuli-Venezia Giulia: Val Cimoliana, 46.391 N 12.48 E, 1700, Ruth Flatscher, 7-Jul-2011

C, montane, PRJNA308183, VCI12-VCI15-VCI20-VCI21-VCI22-VCI23-VCI24-VCI26-VCI27-VCIC, SRR3096562-SRR3096563-SRR3096564-SRR3096565-SRR3096566-SRR3096567-SRR3096568-SRR3096569-SRR3096570-SRR3096571, Italy: Friuli-Venezia Giulia: Val Cimoliana, 46.38 N 12.489 E, 1180, Ruth Flatscher, 7-Jul-2011

D, alpine, PRJNA308183, PHO5-PHO7-PHO11-PHO13-PHO15-PHO16-PHO2-PHO22-PHO23-PHO30, SRR3096528-SRR3096529-SRR3096530-SRR3096531-SRR3096534-SRR3096532-SRR3096533-SRR3096535-SRR3096536-SRR3096537, Austria: Kärnten: Lienzer Dolomiten, 46.762 N 12.877 E, 2055, Ruth Flatscher, 3-Aug-2011

D, montane, PRJNA308183, VHO6-VHO11-VHO13-VHO16-VHO17-VHO18-VHO19-VHO21-VHO25-VHO29, SRR3096584-SRR3096585-SRR3096587-SRR3096593-SRR3096595-SRR3096596-SRR3096597-SRR3096598-SRR3096599-SRR3096600, Austria: Kärnten: Lienzer Dolomiten, 46.774 N 12.901 E, 790, Ruth Flatscher, 3-Aug-2011

E, alpine, PRJNA308183, PNE6-PNE11-PNE14-PNE15-PNE16-PNE19-PNE21-PNE25-PNE26-PNE28, SRR3096539-SRR3096540-SRR3096541-SRR3096542-SRR3096543-SRR3096544-SRR3096546-SRR3096547-SRR3096548-SRR3096549, Italy: Friuli-Venezia Giulia: Alpi Giulie, 46.376 N 13.459 E, 1820, Ruth Flatscher, 6-Jul-2011

E, montane, PRJNA308183, VNE2-VNE20-VNE22-VNE24-VNE25-VNE5-VNE67-VNE73-VNE74-VNE81, SRR3096606-SRR3096602-SRR3096603-SRR3096604-SRR3096605-SRR3096607-SRR3096608-SRR3096609-SRR3096610-SRR3096611, Italy: Friuli-Venezia Giulia: Alpi Giulie, 46.388 N 13.459 E, 1170, Ruth Flatscher, 6-Jul-2011

F, alpine, PRJNA308183, PDI10-PDI16-PDI17-PDI19-PDI20-PDI21-PDI24-PDI25-PDI26-PDI7, SRR3096514-SRR3096517-SRR3096518-SRR3096519-SRR3096520-SRR3096521-SRR3096522-SRR3096523-SRR3096524-SRR3096526, Slovenia: Primorska: Trnovski gozd, 45.989 N 13.845 E, 1100, Božo Frajman, 17-Jul-2011

F, montane, PRJNA308183, VDI4-VDI8-VDI11-VDI25-VDI27-VDI29-VDI3-VDI30-VDIM3-VDIM7, SRR3096573-SRR3096574-SRR3096575-SRR3096576-SRR3096578-SRR3096577-SRR3096579-SRR3096580-SRR3096581-SRR3096582, Slovenia: Primorska: Idrijca valley, 46.117 N 13.911 E, 540, Božo Frajman, 17-Jul-2011

Notes S2. Estimating the proportion of transposable elements in the *Heliosperma* RADseq dataset

In order to detect significant differences in TE abundance between populations of alpine and montane ecotypes of *H. pusillum* we used all RADseq paired-end reads of each individual and the database of transposable elements identified in plants and collected in the RepBase database on the Giri repository (http://www.girinst.org/). We downloaded as a plain fasta file all elements matching “Viridiplantae” in the database, including transposable elements, simple repeats, pseudogenes and integrated viruses. We then indexed this file and separately blasted to this reference all paired-end reads for each individual sample using a maximum e-value of 0.0001. For each individual, we estimated the proportion of reads with successful hit to the TE database. In each ecotype pair, a Kolmogorov-Smirnov test (*R* function *ks.test* in the *stats* package) was implemented to test for significant differentiation in the proportion of TEs. We then grouped the successful hits by TE family and counted the occurrence of each family in each individual. We then selected 11 families of TEs that were found in each of the 120 individuals. After grouping the individuals by ecotype, we assessed the difference in the presence of each family between the two ecotypes, again with a Kolmogorov-Smirnov test.

The percentage of paired-end reads mapping to the Viridiplantae TE database (Fig. S1) was in general low for all individuals spanning from 0.46% (21,757 hits) to 4.1% (45,489 hits). At the level of ecotype pairs a significantly higher proportion of TE hits in the montane ecotype as compared to the alpine one was found in HO and DI ($p < 0.05$) although not passing the Bonferroni corrected threshold (0.008). Across all six ecotype pairs more individuals with higher proportion of hits to transposable elements were found in the montane ecotype (p-value = 0.047, Fig. S2). Gypsy and Copia (Class I Retrotransposons, order LTR) were the most represented superfamilies and more abundant in the montane ecotype, although the difference was not significant (Fig. S2). Retrotransposons belonging to the order LINE, superfamily L1, appeared instead to be significantly more represented in the montane individuals.

Our results exclude any significant bias in our dataset due to RAD loci representing TEs. As our RADseq dataset was enriched for genes (42% of the loci) and genomic clusters of genes are generally poor in TEs (Schmidt and Heslop-Harrison 1998), the low proportion of reads mapping to TEs is not surprising. Although not significant, we found a slightly higher proportion of transposable elements in two out of six montane populations and a general increase in the amount of retrotransposons, especially Gypsy and Copia, in this ecotype. As expected, these two retrotransposon families were the most represented in our dataset thus driving the global pattern. L1, LINE, elements appear to be significantly more represented in the montane populations. Among Class II DNA Transposons, CATCA elements are more abundant (although not significantly after Bonferroni correction) in the montane populations. This family of TEs has been shown to be involved in intron/exon re-arrangements (*i.e.*, alternative splicing) and in changing regulatory pathways of genes, thus likely being important in the early phases of adaptation (Zabala and Vodkin 2007, Alix et al 2008, Buchmann et al 2014). Being just a by-catch of our RADseq experiment, our screening is only superficially describing TE patterns but it highlights the interest in further investigations focused on TEs and on the role they may play in different stages of populations divergence.

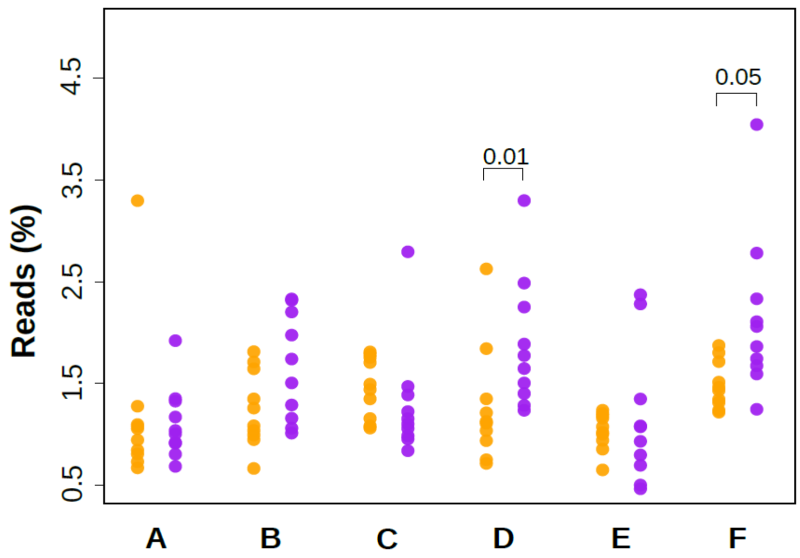

Figure S1. Proportion of transposable elements in 120 individuals of alpine (orange) and montane (purple) *Heliosperma pusillum* grouped in six ecotype pairs. Illustrated are percentages of paired-end reads showing significant (blast e-value < 0.0001) hits to any element listed in the *Giri* database of plant transposable elements. Statistical difference assessed through Kolmogorov-Smirnov test is shown when p-value ≤ 0.05. Bonferroni corrected threshold = 0.008. Population labels as in Fig. 1 in the main text.

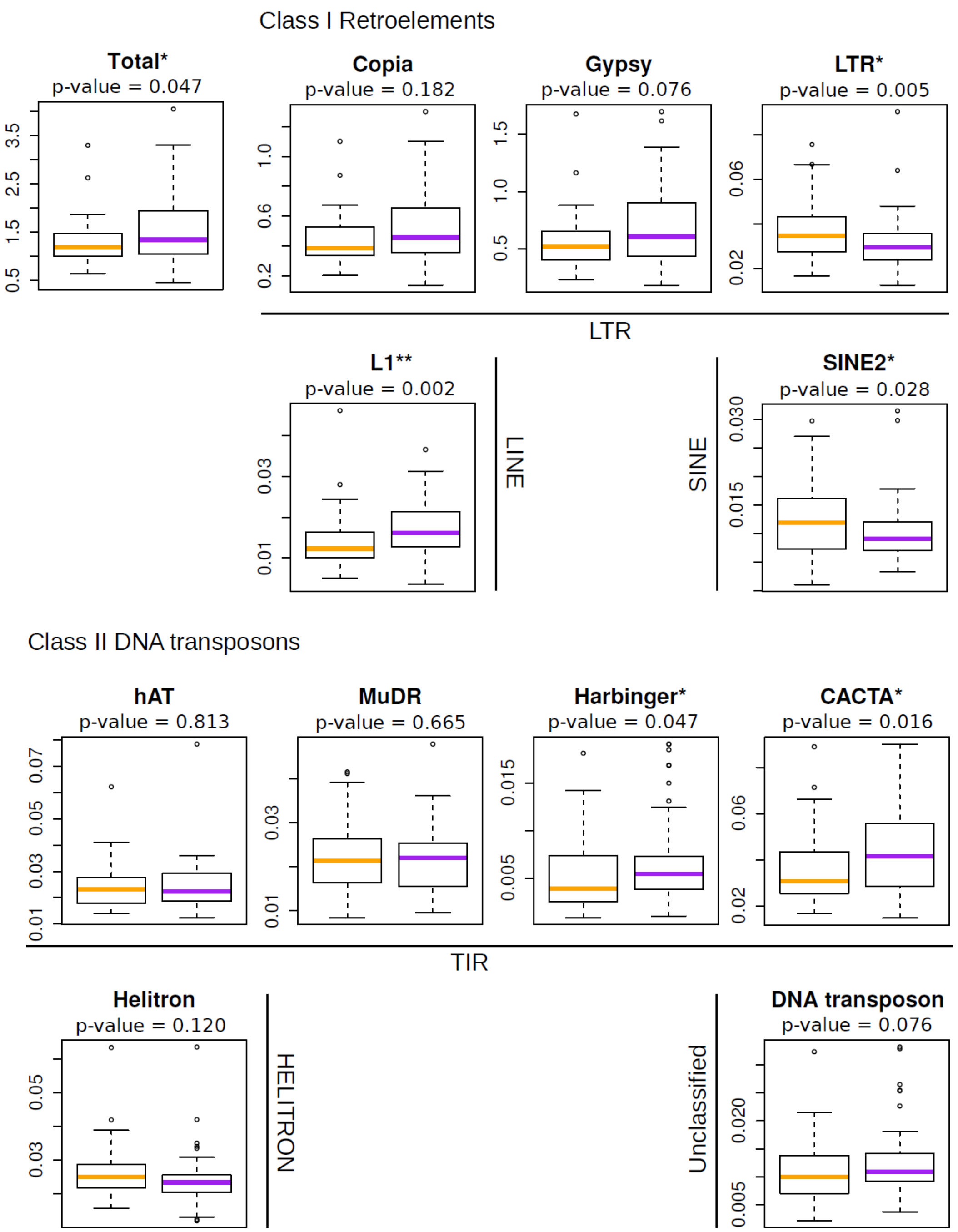

Figure S2. Ecotype-specific proportion of transposable elements in alpine (orange) and montane (purple) individuals of *Heliosperma pusillum*. Boxplots show the percentage of paired-end reads of each individual mapping to plant transposable elements. Superfamilies of transposable elements are grouped in Orders (LTR, LINE, and SINE, and TIR, HELITRON, and Unclassified) within the respective Class (I Retrotransposons and II DNA Transposons). P-values according to Kolmogorov-Smirnov tests are indicated: *p-value < 0.05; **p-value < 0.0045 (Bonferroni correction for 11 comparisons).

References

Alix K, Joets J, Ryder CD, Moore J, Barker GC, Bailey JP, King GJ, Pat Heslop-Harrison JS. 2008. The CACTA transposon Bot1 played a major role in Brassica genome divergence and gene proliferation. *The Plant Journal* **56**: 1030–1044.

Buchmann JP, Löytynoja A, Wicker T, Schulman AH. 2014. Analysis of CACTA transposases reveals intron loss as major factor influencing their exon/intron structure in monocotyledonous and eudicotyledonous hosts. *Mobile DNA* **5**: 24.

Schmidt T, Heslop-Harrison JS. 1998. Genomes, genes and junk: the large-scale organization of plant chromosomes. *Trends in Plant Science* **3**: 195–199.

Zabala G, Vodkin L. 2007. Novel exon combinations generated by alternative splicing of gene fragments mobilized by a CACTA transposon in Glycine max. *BMC Plant Biology* **7**: 38.

Notes S3. Dataset attributes and removal of bacterial contamination

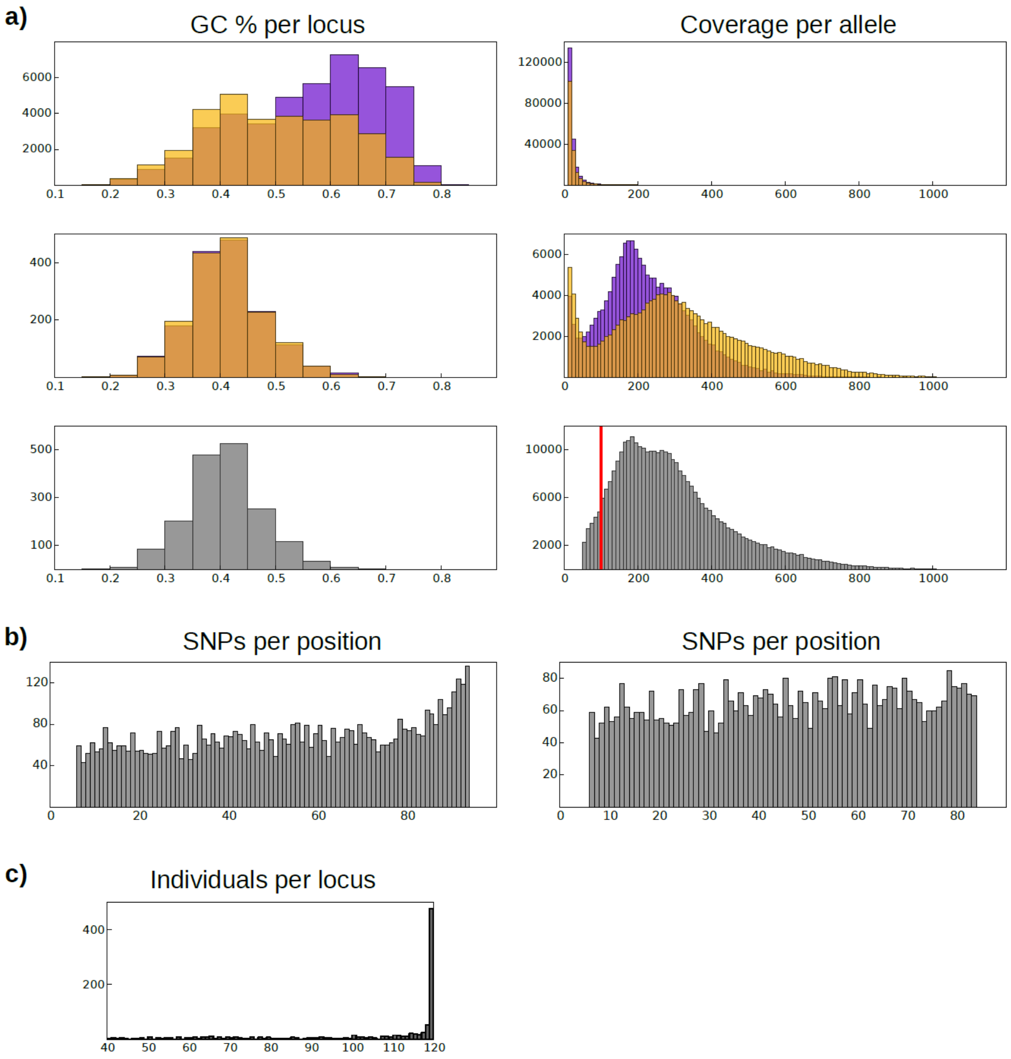

Figure S3. Dataset quality control. a) GC content per locus (left) and coverage per allele (right). Top panel: loci sequenced in two to five individuals; middle panel: loci sequenced in 30 to 60 individuals; bottom panel: loci sequenced in 40 to 120 individuals. Loci assembled in catalogues restricted to alpine and montane individuals are shown in orange and purple, respectively; loci assembled in the final catalog including both ecotypes are in grey. Count of loci is on the y-axis. The threshold for the minimum coverage necessary to call a stacks (-m 100) in the final dataset is shown by a vertical red line. Note that homozygous loci are counted as two alleles and their coverage is divided by two so that alleles showing a coverage lower than the threshold are present in the plot. The difference in the distribution is not due to a library effect as the two ecotypes were sequenced in mixed libraries. b) Distribution of SNPs along the locus length before filtering (left panel) and in the final dataset (right panel) including both ecotypes. Count of SNPs is on the y-axis. c) Number of individuals sequenced per locus in the final dataset. Count of loci is on the y-axis. Custom python script (*loci_selector_v2.py*) employed for filtering the output file generated by the function *export_sql.pl* in the *Stacks* package (see main text for details) is freely available for download from http://www.emilianotrucchi.it/images/loci_selector_v2.py.

Notes S4. Analysis of RADseq Dataset Contamination by Leaf Microbiome

We found a very high proportion of exogenous sequencing reads contaminating the RADseq dataset which were supposed to come from the leaf microbiome (phyllosphere; Vorholt 2012; Bodenhausen et al 2014). We then investigated the composition of the phyllosphere community in the two ecotypes employing the RADseq *metagenomic dataset* (see "*Identification of RAD loci and SNP calling*" in the Materials and Methods). This analysis was performed on different sets of loci that were assembled in *Stacks* and selected in order to identify rare (i.e., occurring in one individual) as well as more common (i.e., occurring in several individuals) contaminant taxa. In order to identify rare vs. common taxa that could be part of the leaf microbiome, we performed a metagenomic analyses on three sets of loci in the *metagenomic dataset* selected to be present in at least one, five, or eight individuals, respectively, in one ecotype at any locality and absent in the other. The analyses were implemented in two steps: *i*) the selected loci were blasted to the NCBI *nt* database using a maximum e-value of 0.0001 and *ii*) the positive hits were summarized using MEGAN (Huson et al. 2007). This software uses an algorithm to assign each hit to the lowest common ancestor (LCA) in the phylogenetic tree (pre-loaded from GenBank). Given the low coverage of contaminants in the RADseq dataset, very conservative thresholds were applied in the LCA algorithm: to include a blast hit in the further analysis, a minimum alignment score of 100 (*min_score* = 100) was required, and to consider a taxon as identified, a minimum number of five hits (*min_support* = 5) had to be assigned to it, or to any of its descendants in the phylogenetic tree. Identified taxa were then summarized at the order level on the phylogenetic tree as identification at lower taxonomic level was considered less reliable. The datasets including loci present in at least five individuals of either ecotypes were further investigated recording number of individuals, coverage per individual, and number of localities in which each locus successfully assigned to a taxon by MEGAN was found; significant differences among populations due to ecology were assessed by PERMANOVA using the function *adonis* in the *R* package *vegan* (Oksanen et al. 2013). As the loci selection we applied could be introducing a bias towards finding differences between the ecotypes, we also searched loci shared by the two ecotypes in each locality selecting loci present in both ecotypes and in at least three individuals in general. Besides confirming that most of the shared loci are actually assigned to the target *Heliosperma* genome, this analysis allowed the identification of locally abundant taxa.

The selection of loci in the RAD dataset that were restricted to either the alpine or the montane ecotype and found in at least one, five or eight individuals were 184,154 and 243,674, 2,301 and 2,879, and 437 and 590, respectively. In the metagenomic analysis, only 20% of the loci were taxonomically assigned in the datasets including loci found in at least one individual, whereas between 42 and 50% were identified in the other two datasets (Fig. S4). In the datasets of loci found in at least five individuals of either ecotype (2,301 and 2,879 in the alpine and montane ecotype, respectively), we found a significant difference in the phyllosphere between the two ecotypes (PERMANOVA, 9999 permutations: $F = 18.8$, p-val = 0.002) with a higher proportion of loci assigned to bacteria and fungi in the montane one (Table S2, Fig. S5). In the alpine ecotype, the bacterial community was characterized by the presence of Cytophagales, Sphingomonadales and Pseudomonadales, whereas Enterobacteriales and Xanthomonadales were only found in the montane one. Bacteria from Rhizobiales and Actinomycetales were found in both ecotypes although the latter were more taxonomically diverse in the alpine individuals. Fungal taxa from Dothideomycetidae, Sordariomycetes and superclass Leotiomycetes were found only in the montane ecotype (Table S2). The structure of the whole phyllosphere community was mostly consistent across all ecotype pairs. Loci assigned to the Xanthomonadales, in particular, co-occurred across most of the montane populations (Table S2). By contrast, very few taxa were shared between the two ecotypes in each pair (Fig. S6). A difference in the number of loci assigned to flowering plant species (summarized as Mesangiospermae) was found between the two ecotypes: 120 loci in the alpine and seven in the montane (Fig. S5, Table 1 in main text). Of the private loci found in the alpine, 108 mapped to plastid sequences whereas none of the seven loci private to the montane mapped to any

plastid sequence. Assuming a neutral accumulation of substitutions at the RADseq restriction enzyme cut-site, we expected a nearly equal proportion of allele drop-out in the two ecotypes (Gautier *et al.* 2013) and, hence, a similar proportion of private loci in the two ecotypes. The discrepancy in our results may be explained by a higher plastid genome proportion in the DNA extracted from alpine individuals. An alternative explanation is related with the highly dynamic and massive mitochondrial genome found in several species of the closely related genus *Silene* (Sloan *et al.* 2014, Wu *et al.* 2015a). This mitochondrial genome has been described as fragmented in multiple circular chromosomes that can include several fast-evolving inserted copies of nuclear and plastid genes (Sloan & Wu 2014), and long intergenic regions that are actively transcribed and may have a regulatory activity (Wu *et al.* 2015b). More dedicated investigations are needed to confirm any difference in mitochondrial genome dynamics and structure between the two ecotypes of *H. pusillum.* We also found a significant component of loci assigned to Diptera (in particular *Drosophila*) in four alpine populations (Figs. S5, Tab. S2). As *Drosophila* clearly prefers humid environments, it is not surprising it was only found on in alpine wet habitat and not on the dry montane one. Why there is much less *Drosophila* contamination in E and F is less clear (all populations were sampled during the same summer season in July to August 2011). Further investigations are needed to identify the *Drosophila* species and clarify this aspect.

We report here preliminary evidence of similarity in the phyllosphere community across different populations of the same ecotype contrasted by parallel differentiation between ecotypes in each locality (Tab. S2, Figs. S4 and S5). Although based on loci found in a few individuals, this result is not surprising as ecological, morphological and genetic divergence in plants may also be associated with consistent changes in the microbiome both at the level of the rhizosphere and, especially, the phyllosphere (Vorholt 2012; Bodenhausen *et al.* 2014). A different set of relationships with a novel range of microbial and fungal organisms can cause a feedback between the host plant and the associated microbial community (Bonfante & Anca 2009; Lebeis 2014) fostering further divergence between populations thriving in contrasting habitats (Margulis & Fester 1991; Thomson 1999). Bacterial and fungal organisms, including commensal, mutualistic and pathogenic taxa living on roots and leaves, have been shown to have a deep physiological, functional and evolutionary impact on the host plant species (e.g., Guttman *et al.* 2014; Kembel *et al*. 2014; Lebeis 2015) and, consequently, on ecological adaptation and evolution (Margulis & Fester 1991; Zilber-Rosenberg & Rosenberg 2008). Including the analysis of the microbiome in molecular ecology studies has been identified as one of the main priorities in the field (Andrew *et al.* 2013), especially through the serendipitous analysis of microbiome-associated contamination of host genome-wide sequencing (Kumar & Blaxter 2012). Here, we show that a high-coverage RADseq dataset can serve as preliminary screen of the phyllosphere of target species. Follow-up investigations should focus on *i*) the identification of microbial taxa in the phyllosphere of both *H. pusillum* ecotypes through in-depth metagenomic analyses, *ii*) the characterization of *Heliosperma*-specific microbiomes (both in phyllosphere and rhizosphere) contrasted with the microbiomes of accompanying plant species, thereby allowing for disentangling the habitat-specific microbial background from *Heliosperma*-specific taxa, and *iii*) the characterization of the functional aspects of the most important biotic interactions.

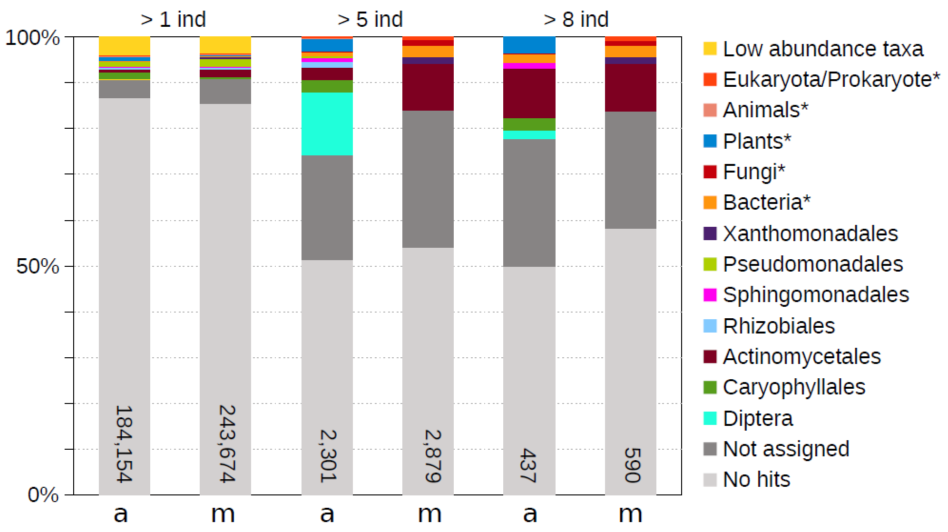

Figure S4. Proportion of loci blasting to the *nt* NCBI database and assigned to order level taxa in MEGAN (see the main text for details and reference) in three nested sets of loci selected as present in at least 1, 5, 8 individuals, respectively, of either alpine or montane individuals of *H. pusillum* (private loci of each ecotype). Loci assigned to higher than order-like level taxa are also reported (asterisk). The number of loci included in each set are shown (a: alpine ecotype; m: montane ecotype).

Table S2. Number, occurrence across individuals, average coverage per individual, occurrence across populations (mean, min and max in brackets) of loci from alpine (a) and montane (m) populations of *H. pusillum* assigned to order-level taxa as well as to higher-than-order-level taxa (marked by an asterisk) by MEGAN after blasting to the *nt* NCBI database. Number of loci present in each population are also shown. a: alpine ecotype; m: montane ecotype. Analyses are performed using the datasets of loci retrieved in at least five individuals (bars 3 and 4 in Figure S4). Population labels are as in Figure 1 in the main text.

	Ecotype	Loci sum	Individual occurrences	Coverage occurrences	Population occurrences	Loci per population A	B	C	D	E	F
Actinomycetales	a	63	9.3(5-14)	149(20-1075)	1.8(1-4)	11	13	8	7	11	63
	m	293	6.3(5-15)	54(20-870)	3.5(2-6)	71	221	212	167	137	209
Cytophagales	a	7	6.3(5-11)	55(21-162)	1.3(1-2)	1	0	1	0	0	7
	m	0	0	0	0	0	0	0	0	0	0
Rhizobiales	a	26	5.4(5-8)	49(20-418)	2.5(1-4)	10	8	16	5	1	25
	m	9	6.6(5-9)	48(20-219)	3.4(2-4)	1	7	9	3	9	2
Sphingomonadales	a	20	6.8(5-12)	62(20-430)	1.6(1-3)	2	3	4	2	1	20
	m	0	0	0	0	0	0	0	0	0	0
Enterobacteriales	a	0	0	0	0	0	0	0	0	0	0
	m	6	5.3(5-6)	45(20-295)	2.5(2-3)	2	1	3	4	2	3
Pseudomonadales	a	11	5.1(5-6)	59(21-245)	2.2(1-3)	4	0	6	1	2	11
	m	0	0	0	0	0	0	0	0	0	0
Xanthomonadales	a	0	0	0	0	0	0	0	0	0	0
	m	42	6.5(5-12)	46(20-272)	4.2(3-6)	8	39	41	19	27	41
Leotiomycetes*	a	5	6.4(5-11)	50(23-162)	3.6(3-4)	4	1	1	4	4	4
	m	10	6.4(5-10)	48(21-163)	3.5(3-5)	6	4	8	8	3	6
Dothideomycetidae	a	0	0	0	0	0	0	0	0	0	0
	m	6	7.8(5-10)	52(23-121)	3.2(3-4)	3	0	6	3	2	5
Sordariomycetes	a	0	0	0	0	0	0	0	0	0	0
	m	7	5.1(5-6)	43(22-288)	3.0(1-4)	1	4	7	3	1	5

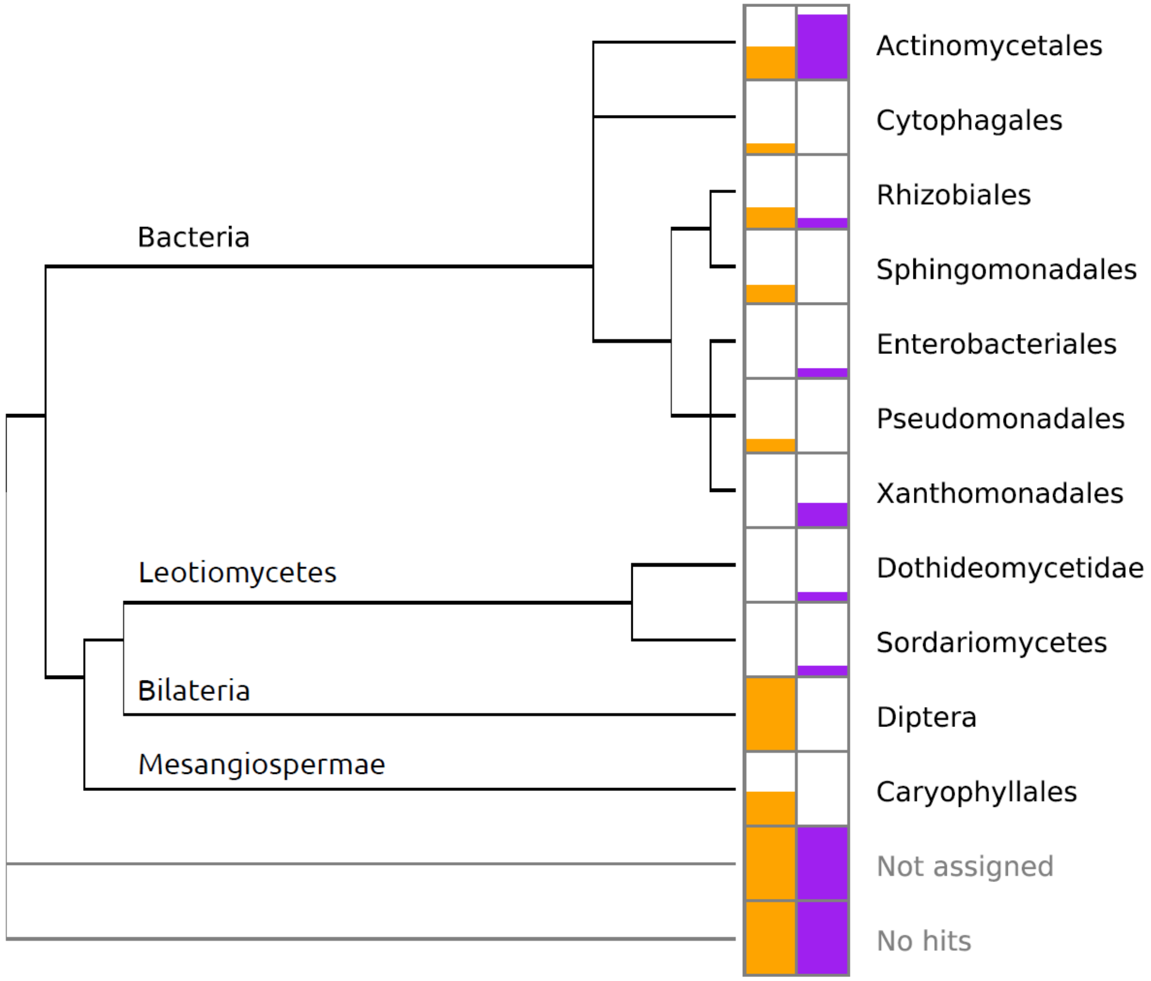

Figure S5. Normalized proportion of loci retrieved from populations of alpine (orange) and montane (purple) *Heliosperma pusillum* blasting to the *nt* NCBI database and assigned to order level taxa arranged on a phylogenetic tree (automatically downloaded from the NCBI database) by MEGAN. Loci without hits to *nt* NCBI database or not assigned to a taxon by MEGAN are also shown.

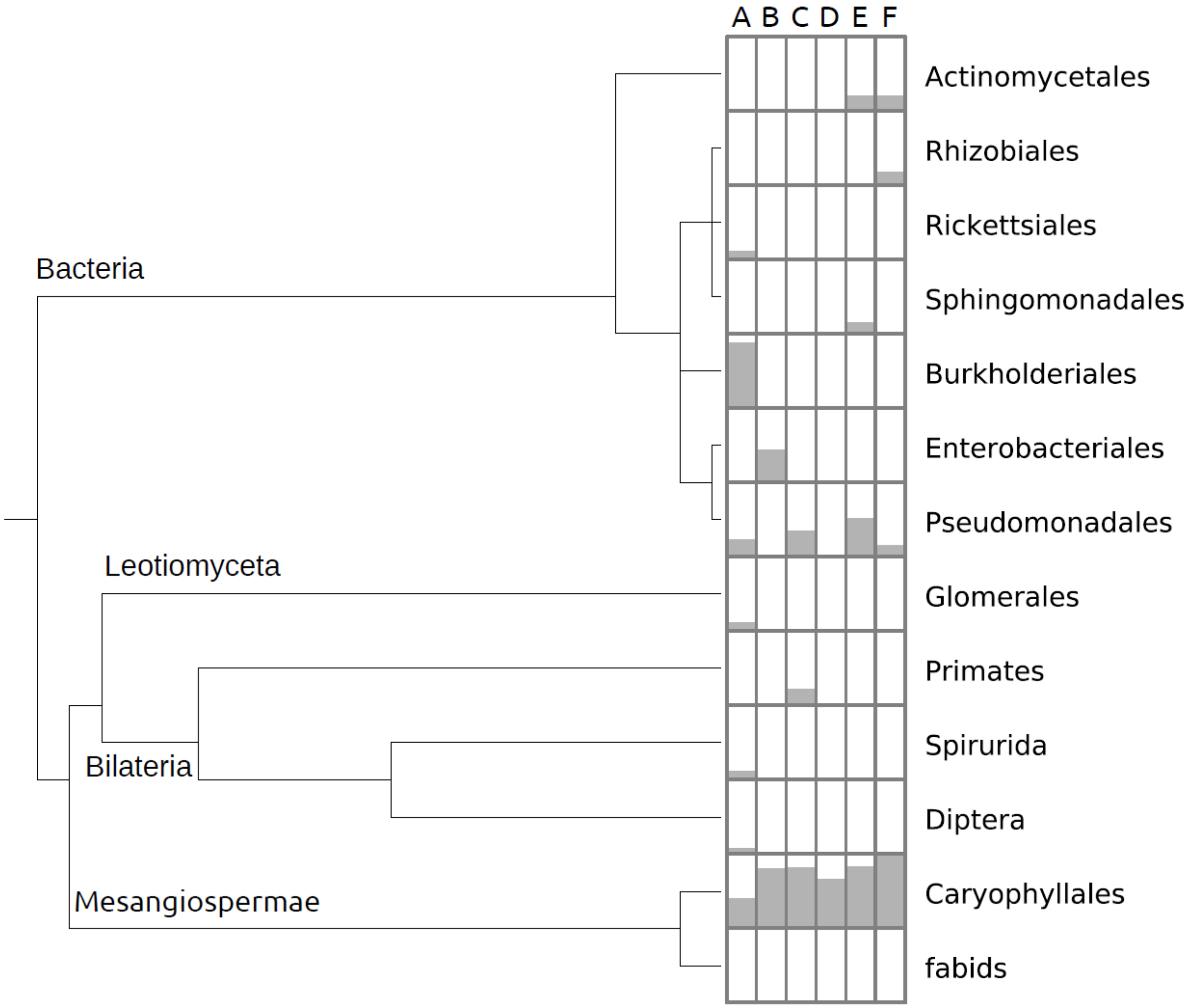

Figure S6. Shared loci between alpine and montane individuals of *Heliosperma pusillum* in each ecotype pair. Loci were selected and retained for blasting if they are present in at least three individuals across the two ecotypes in each pair. Most of the loci were expected to come from the target *Heliosperma* genome and not to be contaminants. Indeed, Caryophyllales (and all the higher hierarchy taxa up to Eukarya not shown here) are well represented by assigned hits. Very little of the exogenous DNA contamination is shared between the two species in each population pair. Taxa with the highest number of hits were *Variovorax paradoxus* in A, the only species contributing to the high prevalence of Burkholderiales in this locality, *Pseudomonas syringae* in A, *P. fluorescens* and *P. trivialis* in E, and *Rahnella aquatilis*, a quite rare Enterobacteriales, in B. Phylogenetic tree and nomenclature as automatically downloaded from GenBank NCBI database by MEGAN.

References

Andrew RL, Bernatchez L, Bonin A, Buerkle CA, Carstens BC, Emerson BC, Garant D, Giraud T, Kane NC, Rogers SM *et al.* 2013. A road map for molecular ecology. *Molecular Ecology* **22:** 2605–2626.

Bodenhausen N, Bortfeld-Miller M, Ackermann M, Vorholt JA. 2014. A Synthetic Community Approach Reveals Plant Genotypes Affecting the Phyllosphere Microbiota. *PLoS Genet* **10:** e1004283.

Bonfante P, Anca I-A. 2009. Plants, Mycorrhizal Fungi, and Bacteria: A Network of Interactions. *Annual Review of Microbiology* **63:** 363–383.

Gautier M, Gharbi K, Cezard T, Foucaud J, Kerdelhué C, Pudlo P, Cornuet JM, Estoup A. 2013. The effect of RAD allele dropout on the estimation of genetic variation within and between populations. *Molecular Ecology* **22:** 3165–3178.

Guttman DS, McHardy AC, Schulze-Lefert P. 2014. Microbial genome-enabled insights into plant-microorganism interactions. *Nature Reviews Genetics* **15:** 797–813.

Huson DH, Auch AF, Qi J, Schuster SC. 2007. MEGAN analysis of metagenomic data. *Genome Research* **17:** 377–386.

Kembel SW, O'Connor TK, Arnold HK, Hubbell SP, Wright SJ, Green JL. 2014. Relationships between phyllosphere bacterial communities and plant functional traits in a neotropical forest. *Proceedings of the National Academy of Sciences* **111:** 13715–13720.

Kumar S, Blaxter ML. 2012. Simultaneous genome sequencing of symbionts and their hosts. *Symbiosis* **55:** 119–126.

Lebeis SL. 2014. The potential for give and take in plant–microbiome relationships. *Frontiers in Plant Science*, **5**: 287.

Lebeis SL. 2015. Greater than the sum of their parts: characterizing plant microbiomes at the community-level. *Current Opinion in Plant Biology* **24:** 82–86.

Margulis L, Fester R. 1991. *Symbiosis as a Source of Evolutionary Innovation: Speciation and Morphogenesis*. Cambridge, USA: MIT Press.

Oksanen J, Blanchet FG, Kindt R, Legendre P, Minchin PR, O'Hara RB, Simpson GL, Solymos P, Stevens MH, Wagner H. 2013. "Vegan: Community Ecology Package. R-package version 2.0-10." *URL http://CRAN. R-project. org/package= vegan*.

Sloan DB, Triant DA, Forrester NJ, Bergner LM, Wu M, Taylor DR. 2014. A recurring syndrome of accelerated plastid genome evolution in the angiosperm tribe Sileneae (Caryophyllaceae). *Molecular Phylogenetics and Evolution* **72:** 82–89.

Sloan DB, Wu Z. 2014. History of plastid DNA insertions reveals weak deletion and at mutation biases in angiosperm mitochondrial genomes. *Genome Biology and Evolution* **6:** 3210–3221.

Thompson JN. 1999. The evolution of species interactions. *Science* **284:** 2116–2118.

Vorholt JA. 2012. Microbial life in the phyllosphere. *Nature Reviews Microbiology* **10:** 828–840.

Wu Z, Cuthbert JM, Taylor DR, Sloan DB. 2015a. The massive mitochondrial genome of the angiosperm *Silene noctiflora* is evolving by gain or loss of entire chromosomes. *Proceedings of the National Academy of Sciences* **112:** 10185–10191.

Wu Z, Stone JD, Štorchová H, Sloan DB. 2015b. High transcript abundance, RNA editing, and small RNAs in intergenic regions within the massive mitochondrial genome of the angiosperm *Silene noctiflora*. *BMC Genomics* **16:** 938.

Zilber-Rosenberg I, Rosenberg E. 2008. Role of microorganisms in the evolution of animals and plants: the hologenome theory of evolution. *FEMS Microbiology Reviews* **32:** 723–735.

Notes S5 Custom python code

Custom python script to convert from diploid genotype calls for each individual of one SNPs per locus into population level allele counts. Ex. two lines of input (modifed output of export_sql.pl from the Stacks package) and output files. Homozygote sites can be encoded as eg. T or T/T.

INPUT

```
# Catalog ID	Annotation		Chr	BP	Consensus Sequence	Num Parents		Num Progeny		Num SNPs
	SNPs	Num Alleles		Alleles	Deleveraged		PCI15	PCI16	PCI17	PCI18	PCI19	PCI20
	PCI22	PCI23	PCI24	PCI30	PDI10	PDI16	PDI17	PDI19	PDI20	PDI21	PDI24	PDI25
	PDI26	PDI7	PHO11	PHO13	PHO15	PHO16	PHO22	PHO23	PHO2	PHO30	PHO5	PHO7
	PNE11	PNE14	PNE15	PNE16	PNE19	PNE21	PNE25	PNE26	PNE28	PNE6	PTO12	PTO19
	PTO20	PTO22	PTO25	PTO27	PTO2	PTO5	PTO6	PTO8	PVA11	PVA14	PVA18	PVA3
	PVA4	PVA5	PVA6	PVA7	PVA8	PVA9	VCI12	VCI15	VCI20	VCI21	VCI22	VCI23
	VCI24	VCI26	VCI27	VCIC	VDI11	VDI25	VDI27	VDI29	VDI30	VDI3	VDI4	VDI8
	VDIM3	VDIM7	VHO11	VHO13	VHO16	VHO17	VHO18	VHO19	VHO21	VHO25	VHO29	VHO6
	VNE20	VNE22	VNE24	VNE25	VNE2	VNE5	VNE67	VNE73	VNE74	VNE81	VTO10	VTO11
	VTO14	VTO16	VTO21	VTO23	VTO27	VTO28	VTO29	VTO8	VVA12	VVA15	VVA16	VVA19
	VVA20	VVA23	VVA26	VVA29	VVA4	VVA6

1648		0

TGCAGGTCTTTATTCAATGCTTCTTCTCTTCCCTATGAAAACAAAAGATTATAAACATAATTACTCTTAAGAAATACGGAGTATTTCCA
CTTTA	119	0	1	64,G>T	2	T;G	0	T/T	T	T	T/G	T
	T/G	T/G	G	T/G	T/G	T	T	T	T	T	T	T
	T	T	T	G/T	G/T	T	T/G	G/T	T/T	G	T	G
	G	T	T	T	T	T	T	T	T	T	T	T/T
	T	T	T	T/T	T	T/T	T	T	G/T	G	T	T/T
	T	T	T/G	T	T	T	T	T	T	T	G	T
	G/T	T		G/T	T	T	T	T	T	T	T	T
	T	T	T	T	T	G	T	T	T/G	T	T/T	T/G
	T	T	T	T	T	T	T/T	T	T	T	T	T
	T/T	T/G	T	T	T/T	T	T	T/T	T	G	T	T
	T/T	T	T/T	G	G/T	T/G	T
```

OUTPUT

```
	aC	aF	aD	aE	aB	aA	mC	mF	mD	mE	mB	mA
1648	[13, 7]	[20, 0]	[10, 10]	[20, 0]	[19, 1]	[17, 3]	[14, 4]	[20, 0]	[16, 4]	[20, 0]	[19, 1]	[14, 6]
```

SCRIPT

```
#! /usr/bin/env python

import sys
import os
import pandas as pd
import numpy as np
from random import shuffle
import matplotlib.pyplot as plt
import matplotlib as mpl

def snp_parser(input_file, pop1):
	file = open(input_file, "r")

	#READ THE HEADER AND CREATE THE KEYS'LIST FOR THE DICTIONARIES
	header = file.readline()
	header = header.split("\n")
	header = header[0].split("\t")

	index_pop1 = []
	for sample in pop1:
		index_sample = header.index(sample)
		index_pop1.append(index_sample)

	keys = header[12:len(header)]
	counter_ind_drop = 0
	counter_loci_dropped = 0
	triallelic_discarded = 0
	invariant = 0
```

```
	#CREATE THE MAIN LIST OF LOCI(ROWS-WISE)
	list_count_alleles = []
	list_num_alleles = []
	list_count_each_allele = []
	list_maf = []
	catalog_ID_list = []

	#CREATE A DICTIONARY PER LINE
	while 1:
		line = file.readline()
		if line == "":
			break
		else:
			line = line.split("\n")
			line = line[0].split("\t")
			alt_alleles = line[10]
			pop_alleles = []
			num_snp = int(line[7])
			if len(alt_alleles.split(';')) > 2:
				triallelic_discarded += 1
			else:
				missing_ind = 0
				geno = line[12:len(line)]
				allele_list = []
				index_set_pop = []
				for sample in pop_all:
					index_sample = header.index(sample)
					index_set_pop.append(index_sample)

				for elem in [line[i] for i in index_set_pop]:
					if len(elem) == num_snp:
						allele_list.append(elem)
					elif len(elem) == num_snp*2+1:
						elem = elem.split("/")
						allele_list.append(elem[0])
						allele_list.append(elem[1])
				alleles = set(allele_list)

				if any(len(ind) > num_snp*2+1 for ind in geno):
					continue
				elif len(alleles) == 0: #add here a counter for invariants len(alleles) == 1
					continue
				elif len(alleles) == 1:
					invariant += 1
				else:
					for genotypes in [line[i] for i in index_pop1]:
						if len(genotypes) == num_snp:
							allele = genotypes
							pop_alleles.append(allele)
							pop_alleles.append(allele)
						elif len(genotypes) == num_snp*2+1:
							allele = genotypes.split("/")
							pop_alleles.append(allele[0])
							pop_alleles.append(allele[1])
						else:
							missing_ind += 1
					count_alleles = len(pop_alleles)
					list_count_alleles.append(count_alleles)

					num_alleles = len(alleles)
					list_num_alleles.append(num_alleles)

					count_each_allele = []
					for x in alleles:
						count = pop_alleles.count(x)
						count_each_allele.append(count)
					list_count_each_allele.append(count_each_allele)
					if sum(count_each_allele) == 0:
						maf = np.nan
					else:
						maf = count_each_allele
					list_maf.append(maf)
					catalog_ID = line[0]
					catalog_ID_list.append(catalog_ID)

	print "Check this (number of usable loci):", len(list_count_alleles),"=", len(list_num_alleles),"=", len(list_count_each_allele)
	print "Triallelic loci discarded:", triallelic_discarded
	print "Invariant loci discarded:", invariant
	return list_count_alleles, list_num_alleles, list_count_each_allele, catalog_ID_list, list_maf

aC=[
'PCI15',
'PCI16',
'PCI17',
'PCI18',
'PCI19',
'PCI20',
'PCI22',
'PCI23',
```

```
'PCI24',
'PCI30']
aF=[
'PDI10',
'PDI16',
'PDI17',
'PDI19',
'PDI20',
'PDI21',
'PDI24',
'PDI25',
'PDI26',
'PDI7']
aD=[
'PHO11',
'PHO13',
'PHO15',
'PHO16',
'PHO22',
'PHO23',
'PHO2',
'PHO30',
'PHO5',
'PHO7']
aE=[
'PNE11',
'PNE14',
'PNE15',
'PNE16',
'PNE19',
'PNE21',
'PNE25',
'PNE26',
'PNE28',
'PNE6']
aB=[
'PTO12',
'PTO19',
'PTO20',
'PTO22',
'PTO25',
'PTO27',
'PTO2',
'PTO5',
'PTO6',
'PTO8']
aA=[
'PVA11',
'PVA14',
'PVA18',
'PVA3',
'PVA4',
'PVA5',
'PVA6',
'PVA7',
'PVA8',
'PVA9']
mC=[
'VCI12',
'VCI15',
'VCI20',
'VCI21',
'VCI22',
'VCI23',
'VCI24',
'VCI26',
'VCI27',
'VCIC']
mF=[
'VDI11',
'VDI25',
'VDI27',
'VDI29',
'VDI30',
'VDI3',
'VDI4',
'VDI8',
'VDIM3',
'VDIM7']
mD=[
'VHO11',
'VHO13',
'VHO16',
'VHO17',
'VHO18',
'VHO19',
'VHO21',
'VHO25',
'VHO29',
'VHO6']
```

```
mE=[
'VNE20',
'VNE22',
'VNE24',
'VNE25',
'VNE2',
'VNE5',
'VNE67',
'VNE73',
'VNE74',
'VNE81']
mB=[
'VTO10',
'VTO11',
'VTO14',
'VTO16',
'VTO21',
'VTO23',
'VTO27',
'VTO28',
'VTO29',
'VTO8']
mA=[
'VVA12',
'VVA15',
'VVA16',
'VVA19',
'VVA20',
'VVA23',
'VVA26',
'VVA29',
'VVA4',
'VVA6']

pops = [aC, aF, aD, aE, aB, aA, mC, mF, mD, mE, mB, mA ]
pop_all = aC + aF + aD + aE + aB + aA + mC + mF + mD + mE + mB + mA
pop_names = ['aC', 'aF', 'aD', 'aE', 'aB', 'aA', 'mC', 'mF', 'mD', 'mE', 'mB', 'mA']

pop_num=0
for population in pops:

        list_count_alleles, list_num_alleles, list_count_each_allele, catalog_ID_list, list_maf = snp_parser(sys.argv[1],population)

        if pop_num == 0:
                pop = pop_names[pop_num]
                s = pd.Series(list_count_each_allele, index=[catalog_ID_list])
                foldedSFS = pd.Series(list_maf, index=[catalog_ID_list])
                dataset = pd.DataFrame(s, columns=[pop])
                jointSFS = pd.DataFrame(foldedSFS, columns=[pop])

        else:
                pop = pop_names[pop_num]
                s = pd.Series(list_count_each_allele, index=[catalog_ID_list])
                foldedSFS = pd.Series(list_maf, index=[catalog_ID_list])
                dataset[pop] = pd.DataFrame(s)
                jointSFS[pop] = pd.DataFrame(foldedSFS)

        pop_num +=1

out = sys.argv[1]
out += '_pop_all_freq'
dataset.to_csv(out, header= True, index = True, sep='\t')
```

Notes S6. Testing Alternative Demographic Scenarios

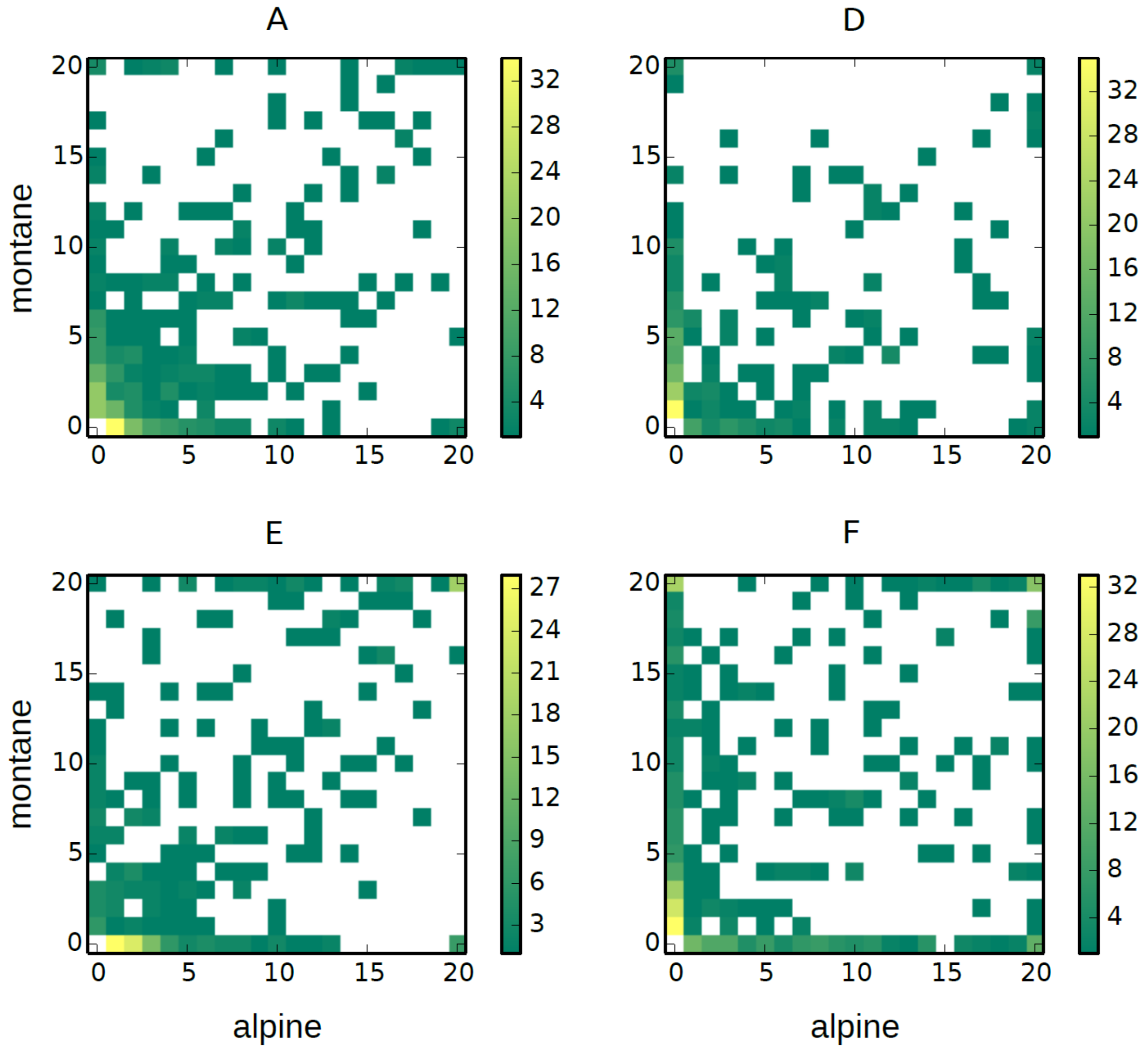

Figure S7. Minor 2D-SFS estimated in each ecotype pair. The minor allele has been identified using the whole dataset (120 individuals from 12 populations) before estimating the minor allele frequency in each population. The frequency of the minor allele (estimated globally) can then be above 0.5 in any particular population (top right quadrant of the plots). This approach for identifying the minor allele and estimating local 2D-SFS is outlined by Excoffier L. in the discussion group on fastsimcoal2 (***https://groups.google.com/forum/#searchin/fastsimcoal/minor$20allele$20/fastsimcoal/zWO_ERhHjOg/3yqgoFZAkZYJ***)

Notes S7. Summary statistics of genomic diversity

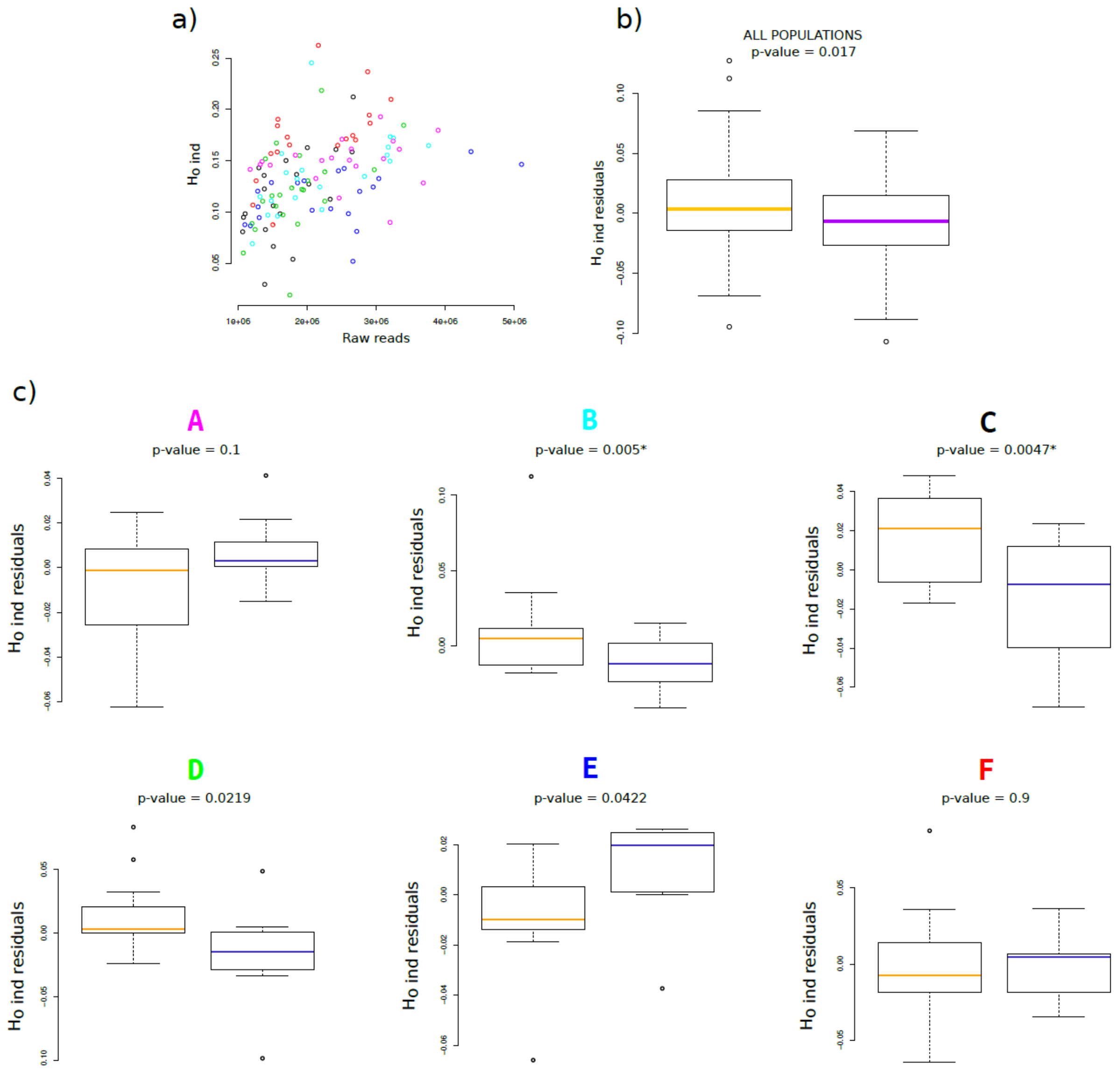

Figure S8. Individual observed heterozygosity normalized by coverage. a) Distribution of average individual observed heterozygosity and sequencing coverage depth in each individual. Colors correspond to panel c. b) Using the coverage as a predictor of individual observed heterozygosity in a linear model, we calculated the deviation of each individual from the model (i.e., analysis of model's residuals) and then plotted the results grouping all of the individuals by ecotype (see Trucchi *et al.* 2016 for details). c) Analysis of model's residual grouping individuals by ecotype in each locality. Statistical significance between ecotypes was assessed by F-test comparing a linear model where observed heterozygosity was predicted by both the number of reads and the ecotype origin of the sample with a reduced model with the number of reads as the only predictor. Significant *p*-value after Bonferroni correction are marked with an asterisk in case of six comparison as in panel c. Orange: alpine ecotype; blue: montane ecotype; population labels as in Fig. 1.

Table S3. Summary statistics of genetic diversity (He: expected heterozygosity; θ: Watterson's θ; π: pairwise differences) in each locality (A to F as in Fig. 2) of the alpine (a) and montane (b) ecotype.

	H_e	θ	π
A_a	0.167	0.239	0.263
A_m	0.161	0.237	0.252
B_a	0.197	0.315	0.323
B_m	0.142	0.199	0.222
C_a	0.165	0.236	0.261
C_m	0.119	0.154	0.173
D_a	0.141	0.199	0.221
D_m	0.100	0.121	0.151
E_a	0.110	0.132	0.163
E_m	0.144	0.204	0.225
F_a	0.196	0.267	0.305
F_m	0.160	0.209	0.258

Notes S8. Structure of genetic diversity

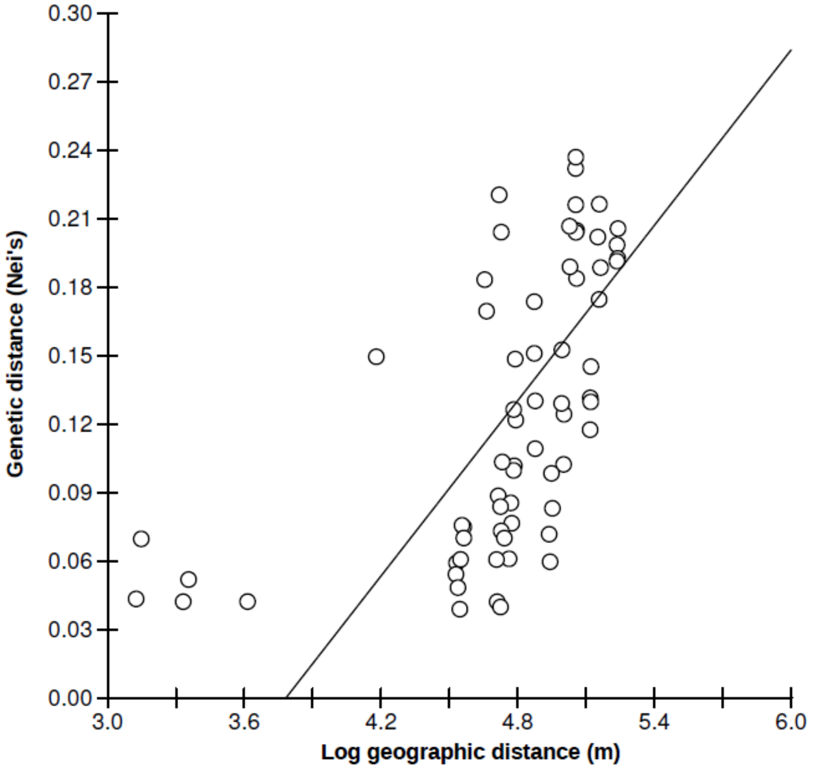

Figure S9. Plot of genetic vs. geographic differentiation testing an isolation-by-distance model. Pairwise distances between alpine and montane *H. pusillum* populations are shown as empty circles and the linear regression is shown as a black solid line.

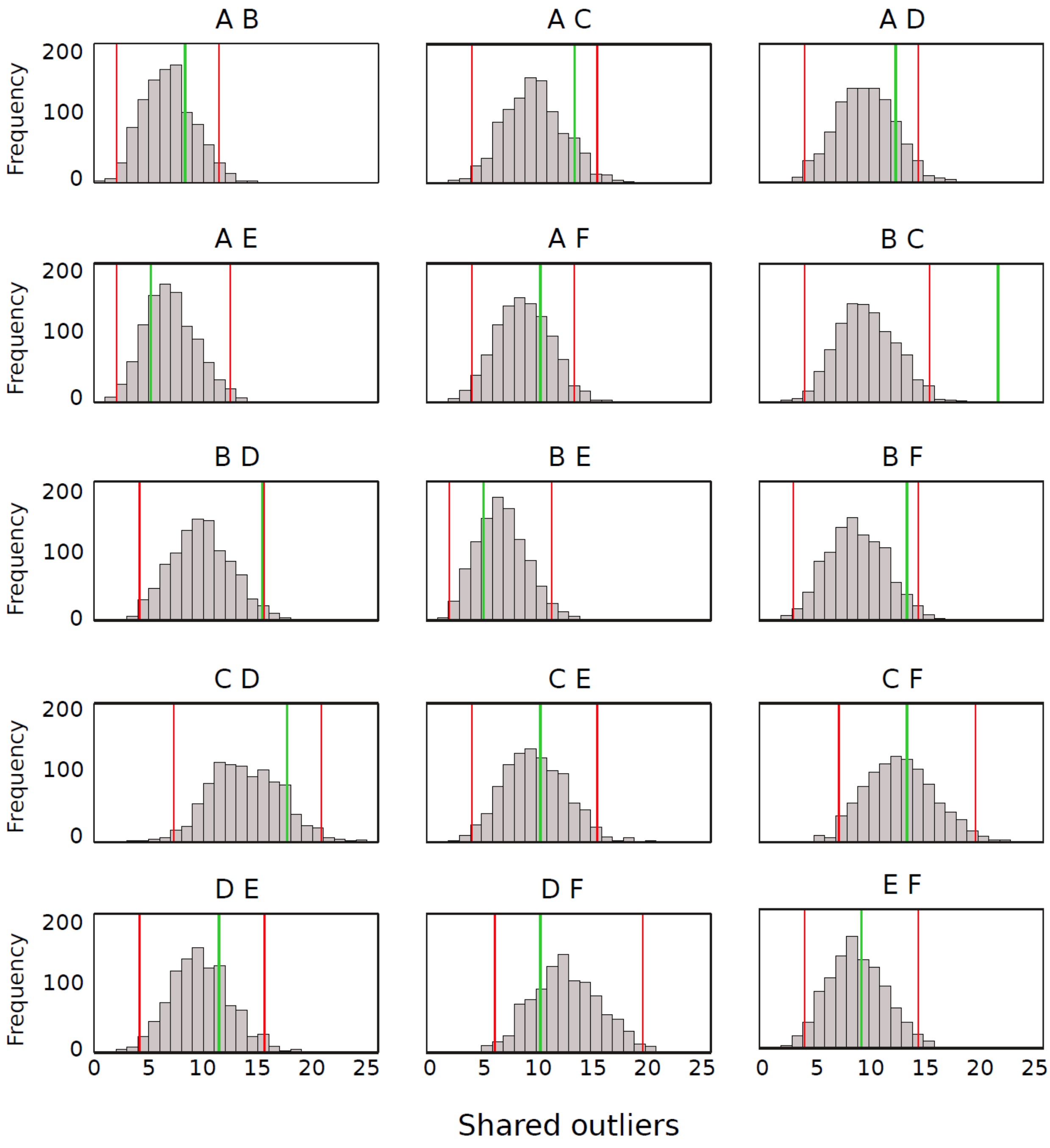

Figure S10. Highly-divergent loci shared among two ecotype pairs (green line). Gray bars: null-distribution (1000 randomizations) under neutrality with 95% quantiles (red line).

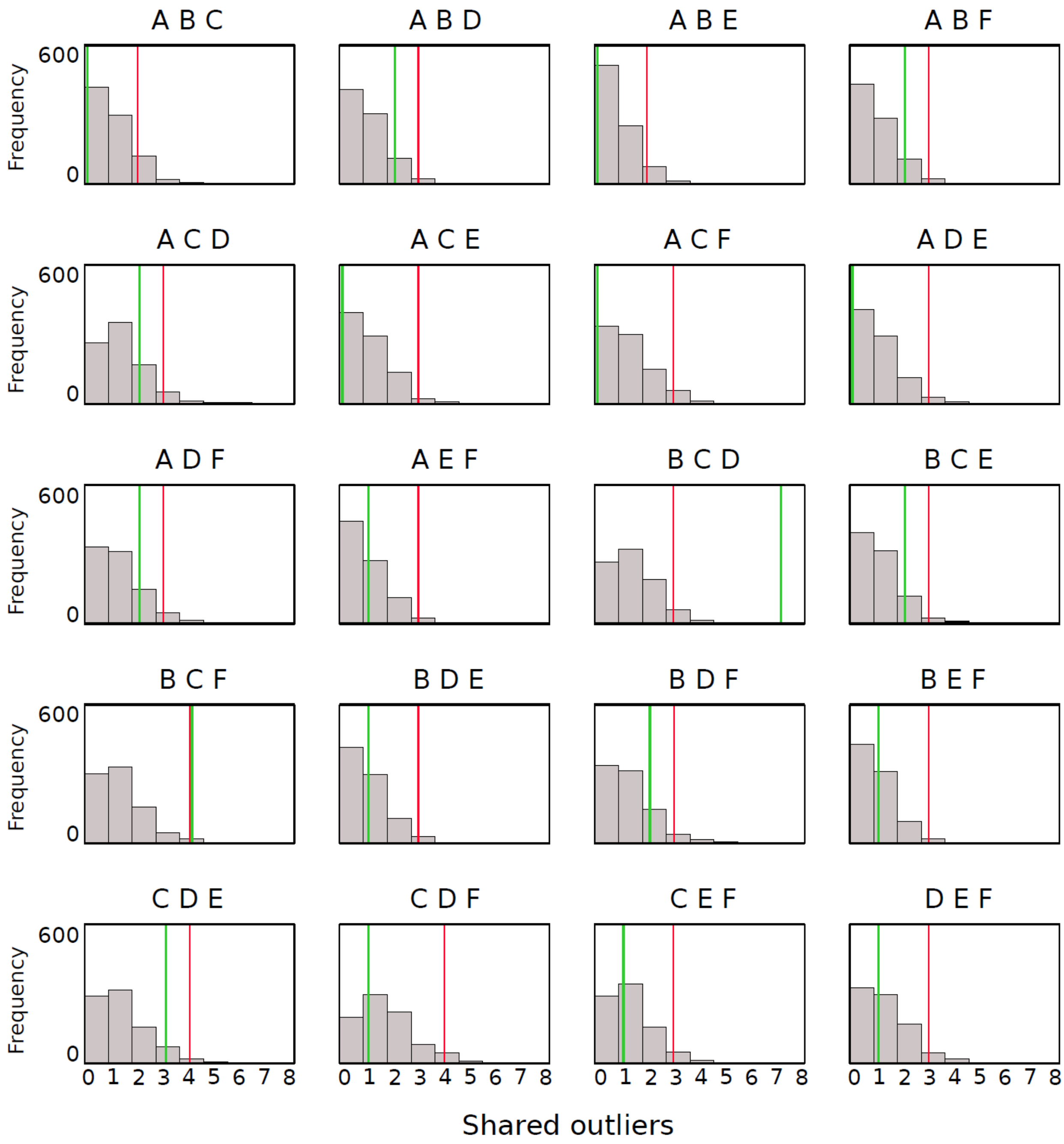

Figure S11. Highly-divergent loci shared among three ecotype pairs (green line). Gray bars: null-distribution (1000 randomizations) under neutrality with 95% quantiles (red line).

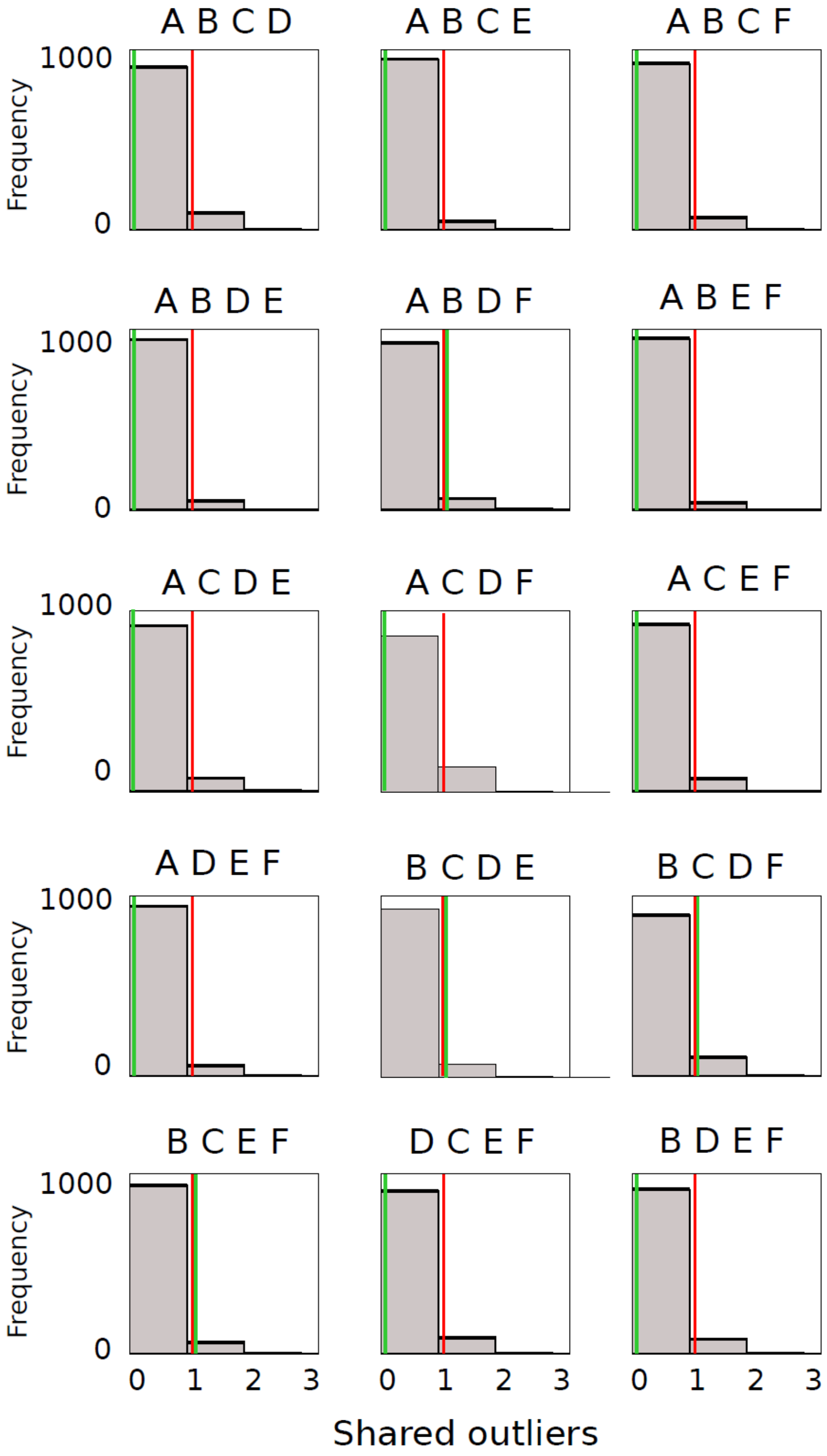

Figure S12. Highly-divergent loci shared among four ecotype pairs (green line). Gray bars: null-distribution (1000 randomizations) under neutrality with 95% quantiles (red line).

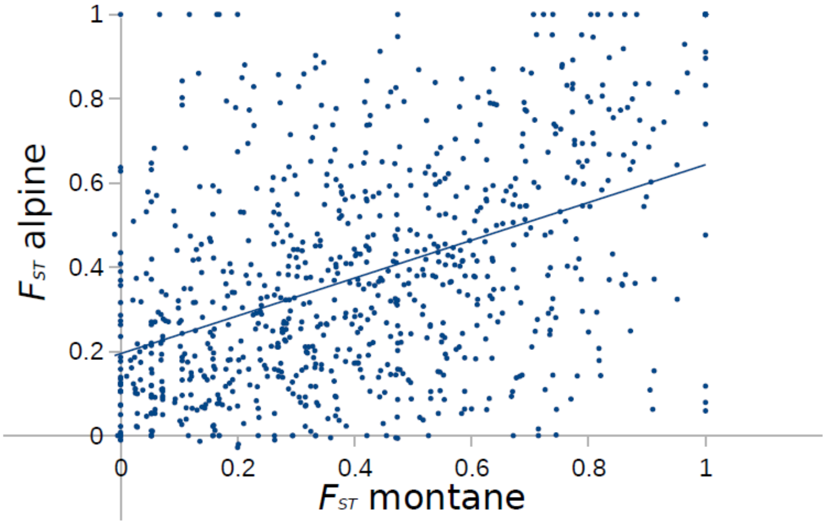

Figure S13. Joint distribution of F_{ST} between alpine and montane populations across all ecotype pairs.

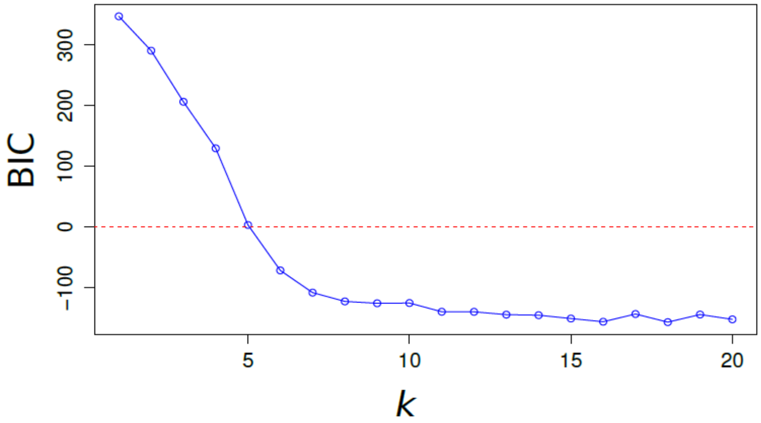

Figure S14. Bayesian Information Criterion of a model describing the structure in the *Heliosperma* populations based on a number of clusters from 1 to 20.

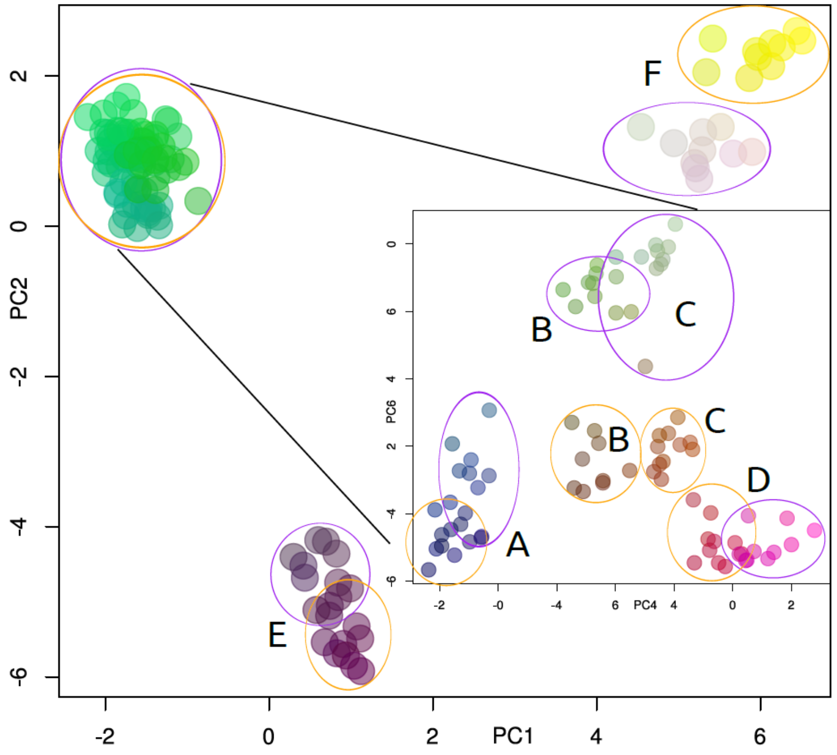

Figure S15. Scatterplot of the first two principal components of a PCA analysis based on 1,097 loci from 120 individuals of alpine (orange) and montane (blue) *H. pusillum*. The circle filling colours were obtained using the first three principal components as separate RGB channels. In the inset, a PCA performed on 80 individuals from population pairs A–D is shown. Population labels as in Figure 1.
